# Rhythms and Community Dynamics of a Hydrothermal Tubeworm Assemblage at Main Endeavour Field – A Multidisciplinary Deep-Sea Observatory Approach

**DOI:** 10.1371/journal.pone.0096924

**Published:** 2014-05-08

**Authors:** Daphne Cuvelier, Pierre Legendre, Agathe Laes, Pierre-Marie Sarradin, Jozée Sarrazin

**Affiliations:** 1 Institut Carnot Ifremer EDROME, Centre de Bretagne, REM/EEP, Laboratoire Environnement Profond, Plouzané, France; 2 Département de Sciences Biologiques, Université de Montréal, succursale Centre-ville, Montréal, Québec, Canada; 3 Institut Carnot Ifremer EDROME, Centre de Bretagne, REM/RDT, Laboratoire Détection, Capteurs et Mesures, Plouzané, France; University of Ferrara, Italy

## Abstract

The NEPTUNE cabled observatory network hosts an ecological module called TEMPO-mini that focuses on hydrothermal vent ecology and time series, granting us real-time access to data originating from the deep sea. In 2011–2012, during TEMPO-mini’s first deployment on the NEPTUNE network, the module recorded high-resolution imagery, temperature, iron (Fe) and oxygen on a hydrothermal assemblage at 2186 m depth at Main Endeavour Field (North East Pacific). 23 days of continuous imagery were analysed with an hourly frequency. Community dynamics were analysed in detail for *Ridgeia piscesae* tubeworms, Polynoidae, Pycnogonida and Buccinidae, documenting faunal variations, natural change and biotic interactions in the filmed tubeworm assemblage as well as links with the local environment. Semi-diurnal and diurnal periods were identified both in fauna and environment, revealing the influence of tidal cycles. Species interactions were described and distribution patterns were indicative of possible microhabitat preference. The importance of high-resolution frequencies (<1 h) to fully comprehend rhythms in fauna and environment was emphasised, as well as the need for the development of automated or semi-automated imagery analysis tools.

## Introduction

The deep sea represents one of the least studied areas of our planet. To date, a mere 5–7% of the deep-sea floor has been explored. Even so, a myriad of unique species and ecosystems have been discovered in the deep with the chemosynthetic environments and their unique fauna as one of the most surprising discoveries of the last 40 years.

Currently, we are still striving to comprehend deep-sea ecosystem functioning, and to fulfil that objective time series are indispensable. To date, hydrothermal vent time series have been mostly based on annual or pluri-annual visits that were carried out with remotely operated vehicles (ROV’s) or submersibles. Due to the peculiarities of the ecosystems and the use of both manned and unmanned submersibles equipped with video cameras, image analysis has always been an important tool to assess deep-sea community and ecosystem changes (see [1] for an overview of studies using imagery at hydrothermal vents). So far, temporal variation studies at hydrothermal vents have enlightened us on successional patterns as well as on communities’ reactions to disturbances, natural or anthropogenic. In post-eruption studies, the nascence of hydrothermal vents is described, in which changes in community dynamics of pioneers and subsequent colonizers were linked with variations in temperature, sulphide supply, and fluid composition [2], [3], [4], [5]. First colonizers tend to tolerate higher temperatures and higher sulphide values while later arrivals live in lower temperature and lower sulphide concentrations. In studies under continuous venting conditions, the de- and reactivation of fluid exits, chimney collapse, and progressive mineralization of hydrothermal edifices contribute to fluid flow modification at small spatial scales, which, by rendering local habitats inhabitable and/or unfavourable, affect faunal distribution and dynamics [6], [7], [8], [9]. As mentioned before, these patterns are mostly deduced from observations separated by a year or more, not recording the cause and effect as it happens, which can only be observed through continuous monitoring. Next to these inter-annual visits, the use of time-lapse cameras at hydrothermal vents (a 26-day deployment at Axial seamount [10] and a 9 month deployment at TAG hydrothermal mound (Mid-Atlantic Ridge (MAR)) [11]) has already demonstrated that sub-annual processes, such as diurnal or semi-diurnal periods, also play a role in shaping hydrothermal vent communities and influence their dynamics and behaviour. High-frequency investigations, with year-round observations, are needed to inform us on natural changes, successional patterns, biotic interactions, reactions to disturbances and ecosystem resilience.

In this 21^st^ century, technology has progressed in such a way that the difficulties of accessing and working in the deep sea are less of a threshold than before. Scientifically, this is illustrated by the development and implementation of deep-sea observatories, operational in a variety of settings [12]. In 2009, a regional-scale cabled observatory network, NEPTUNE (North-East Pacific Time-series Undersea Networked Experiments, an installation of Ocean Networks Canada www.oceannetworks.ca), came online. It consists of an 800 km electro-optic cable loop laid on the seabed over the northern Juan de Fuca tectonic plate, off the coast of British Columbia (Canada), granting real-time access to the data collected by the observatories. An ecological observatory module (TEMPO-mini) equipped with a video camera and environmental probes, is implemented at one instrument node of the NEPTUNE network and focuses on hydrothermal vent ecology. This observatory provides insights in the day-to-day activity of a hydrothermal faunal assemblage and the community dynamics occurring in a natural setting at Main Endeavour Field (MEF). In the present era of deep-sea exploitation, insights in natural change and community resilience are of utmost importance to evaluate the vulnerability and recovery of the hydrothermal vent ecosystems.

MEF was designated as a Marine Protected Area (MPA) in 2003, thus establishing one of the world’s first deep-sea marine protected areas [13]. Its designation as a MPA prevents commercial exploitation, but scientific research is still permitted at specific sites. Nevertheless, sampling is restrained (sampling permits need to be applied for through ‘Fisheries and Oceans Canada’), making imagery an adequate monitoring tool. The video imagery collected by the TEMPO-mini module monitors a *Ridgeia piscesae* tubeworm assemblage that has not been disturbed by sampling. A 23-day period was exhaustively investigated with an hourly frequency to analyse the changes over time documented in the faunal assemblage. Here we describe the (i) community dynamics for all taxa observed as well as their interactions and links with the environment and (ii) rhythms in faunal variation and environmental variables. This multidisciplinary observatory approach informs us on community dynamics and behavioural rhythms of the deep-sea hydrothermal vent fauna and its links with the environment.

## Materials and Methods

### 1. Study Site and Observatory

**Figure 1:**
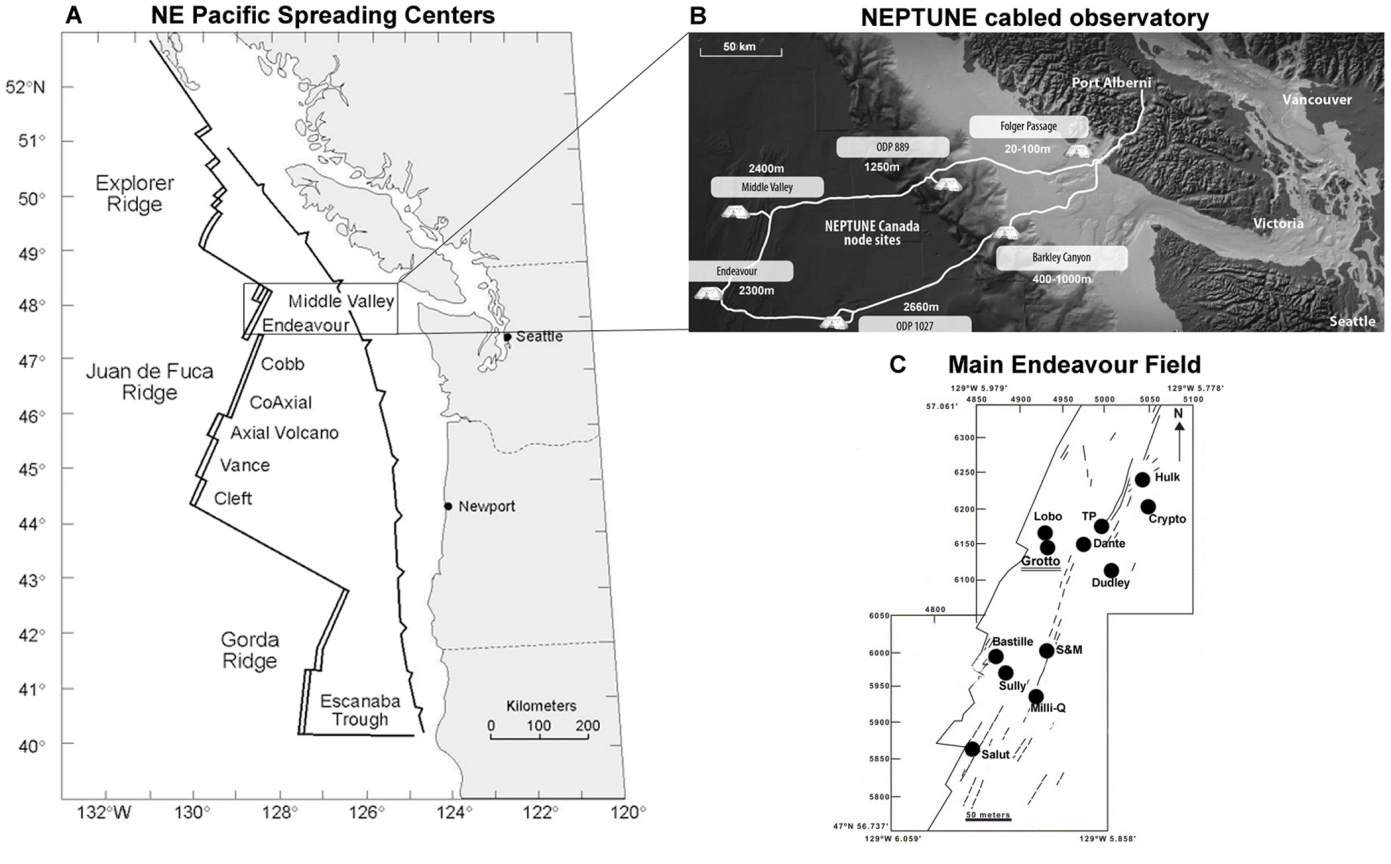
Location and lay-out of NEPTUNE observatory and TEMPO-mini study-site. Location (a) and lay-out (b) of the NEPTUNE cabled observatory on the northern Juan de Fuca plate. The NEPTUNE network contains multiple instrumented nodes (b). The Endeavour node connects the instrument platforms and the instruments at the Main Endeavour Field and contains the TEMPO-Mini module, which has been deployed at Grotto hydrothermal vent (c). MEF map based on [57] and V. Robigou (1995) (unpublished data).

NEPTUNE is a cabled observatory network located in the North-East Pacific off the coast of British Columbia (Canada), which contains various instrumented nodes (Fig. 1). TEMPO-mini is the ecological module [14] that focuses on hydrothermal vent ecology and is connected to the deep-sea Endeavour node (Fig. 1b). This module is the cabled counterpart of the autonomous Atlantic module TEMPO [15], and is used to record imagery and environmental variables in order to study temporal dynamics at deep-sea hydrothermal vents. TEMPO-mini was deployed at the Grotto hydrothermal edifice, at 2186 m depth, within the Main Endeavour Field (MEF, Fig. 1c). The Endeavour segment of the Juan de Fuca spreading ridge (Fig. 1a), and more specifically MEF, has a long history of hydrothermal vent research covering over 25 years [e.g. 7, 16, 17, 18, 19].

The main advantages of a cabled observatory are that energy, battery life and hard drive space are no longer main limiting factors, as opposed to the autonomous, wireless observatories. The instrumented nodes of the NEPTUNE observatory are powered by the on-shore facilities of Port Alberni on Vancouver Island. Thanks to live streaming, data collected is available in real-time on the internet (www.oceannetworks.ca) and is stored and managed at the University of Victoria in British Columbia (Fig. 1b).

### 2. Data Recordings and Observatory Set-up

TEMPO-mini’s first deployment on the NEPTUNE network took place in September 2011 and it was recovered in June 2012; both were carried out with the Remotely Operated Vehicle (ROV) ROPOS. Since its deployment on September 29, 2011, TEMPO-mini transmitted data until the day of its recovery (June 19, 2012). The first week of the deployment was used to run tests in lighting, zoom of the camera, etc., while for the 23 subsequent days (October 7 (8 h UTC) – October 30, 2011 (14 h UTC)), imagery was recorded continuously. Lights were on constantly during the continuous imagery recording period. After these 23 days, the recording frequency changed to one half-hour (30 min) recording every 4 hours and corresponded with a substantial change in zoom (zoom-out, enlarging the filmed surface by a factor of 1.6). From this point onward, the imagery recorded was too zoomed-out to allow the quantitative assessment of macrofaunal densities. An additional problem arose with the oil in which the LED’s were bathed causing a chemical reaction with the lenses of the projectors. This chemical reaction increased opacification of the lights’ lenses which, consequently, emitted less light causing a darkening of the imagery recorded. Still, the module continued to record imagery until its recovery. Camera and light lenses as well as sampling inlets were protected against biofouling through a localised microchloration process [15].

The module was equipped with an Axis Q1755 camera featuring a 1/3″ Progressive Scan CMOS 2 Megapixel image sensor, which recorded videos with a resolution of 1440×1080 pixels and a frame rate of 24 fps. The faunal assemblage filmed was identified as ‘community V low-flow’ [7], characterised by slender *Ridgeia piscesae* tubeworms (Siboglinidae) along with abundant gastropod (Buccinidae, Lepetodrilidae, Provannidae) and pycnogonid fauna.

**Figure 2:**
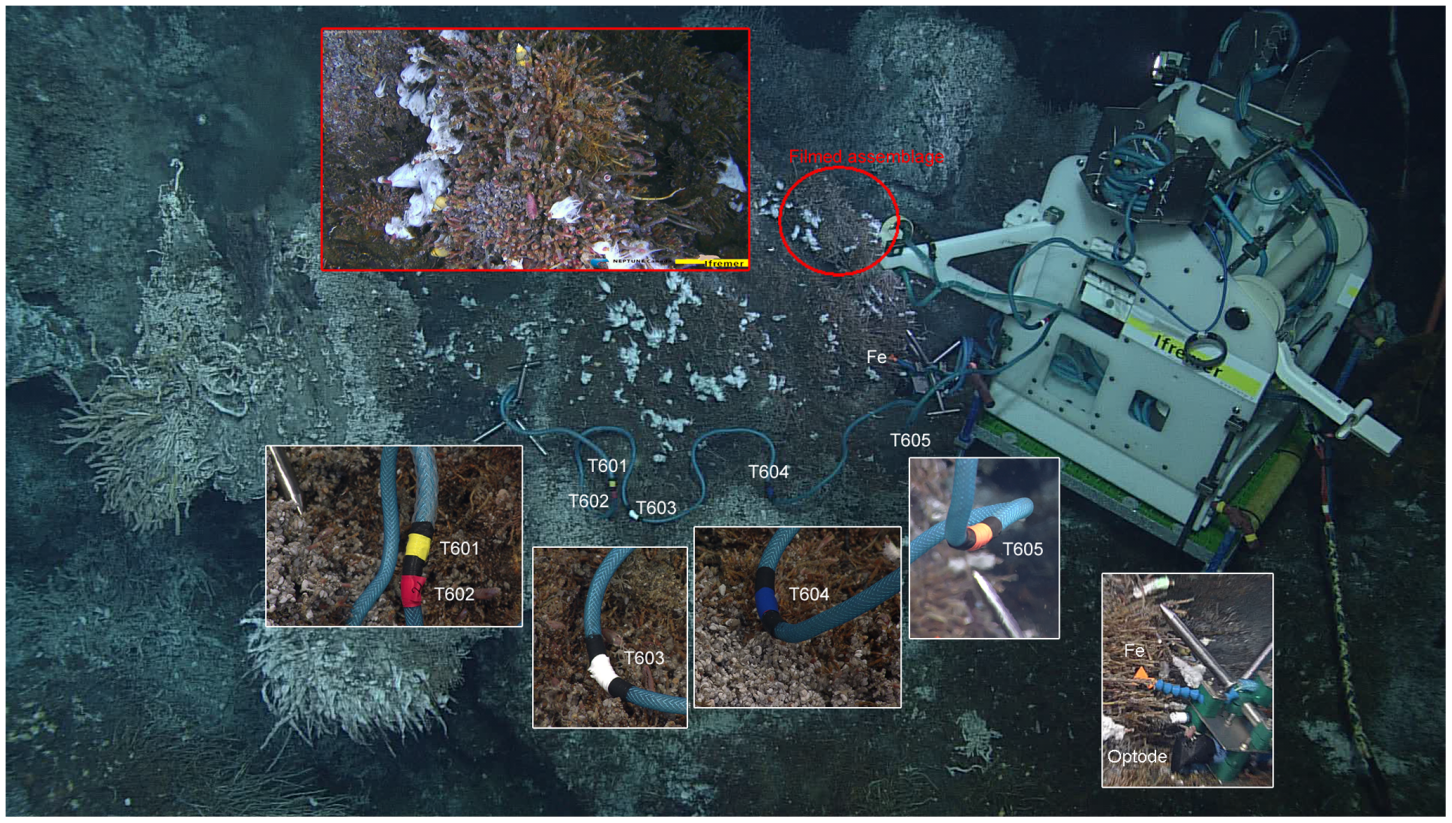
TEMPO-mini module *in situ*. TEMPO-mini *in situ*, at Grotto hydrothermal vent at 2186 m depth. Insets show the filmed assemblage as well as the deployed environmental probes. T60x were the temperature probes and CHEMINI Fe and optode sampling inlets were mounted on a tripod deployed below the filmed assemblage.

A 10 m long thermistor array with 10 temperature probes (separated by less than 1 m) was deployed in the neighbourhood of the filmed assemblage (Fig. 2). Only four temperature probes were positioned on the faunal assemblages at Grotto (T601 to T604); the fifth one (T605) was suspended in mid-water next to the TEMPO-mini module (Fig. 2). Probes T602 and T603 were positioned on assemblages identified as community IV [7], which was considered to be the most similar to the filmed assemblage as the latter (community V) is a subsequent stage of community IV in the succession model proposed by Sarrazin et al. [7]. The ecological framework of the observatory deployment is explained in more detail in the Discussion section. Temperature recordings had a resolution of 1 measurement every 30 seconds. Another tripod with environmental probes was deployed ca. 30–40 cm below the filmed assemblage. It comprised a CHEMINI Fe *in situ* analyser [20], recording three measurement cycles of dissolved iron (referred to as Fe from hereon) every 12 hours and an Anderaa optode, measuring oxygen concentrations (mL/L) and temperature every 30 seconds (Fig. 2).

### 3. Image Analyses

**Figure 3:**
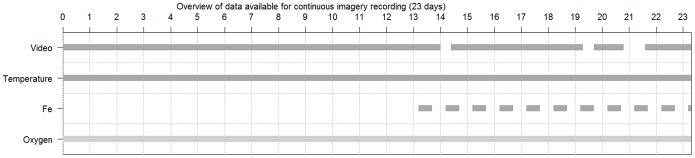
Data overview for continuous imagery duration. Data overview for continuous imagery duration, i.e. 559∼23 days, during which video recording was programmed to be continuous. The gaps in the recordings were unusable video sequences (empty, black or unfocused). Temperature was recorded continuously (1 measurement every 30 seconds). For this period, Fe was measured once a day from October 20, 2011 onward. Oxygen was recorded continuously for the period, although it showed a steep decrease and other inexplicable patterns until day 83, rendering the data unusable (light-grey).

Main focus laid on the analysis of the imagery available for the continuous 23-day period, which featured the most constant zoom and few black-outs, adding up to ∼550 h of video footage (Fig. 3). The surface of the tubeworm bush that was filmed totalled approximately ∼0.0355 m^2^ (ca. 20×18 cm). For this period (2011-10-07 8 h–2011-10-30 14 h), screen-stills were taken hourly, all times are noted in Coordinated Universal Time (UTC). A total of 23 video sequences were either unavailable or unusable (too dark or unfocused) for the predefined hourly frequencies. Overall, 536 hourly screen-stills were used as templates to map and count faunal abundances. During this continuous recording period, the zoom changed twice. The zoom changed only slightly, nevertheless we chose to work with the faunal densities to allow comparisons. Faunal densities were quantified for each hourly image, while for one image every 4 hours the microbial coverage was assessed. To pursue the latter, the microbial cover was marked in white and the rest of the image rendered in black. Using the “magic wand tool” of the ImageJ image analysis software [21], the surface covered by microbes was quantified and converted to percentages.

“Heat maps” were created for all mobile organisms. These maps are a graphical presentation of the numbers of observations using colour-codes. The colours thus reveal the most (dark red) and least (dark blue) frequented areas and distribution patterns for each taxon. For this purpose, the packages gplots [22] and hydroTSM [23], both in R 2.15.1 [24] were used. Several functions were adapted within the packages to meet our requirements and to fit our objectives. Pixel coordinates were attributed to individuals with the ImageJ “point picker” plugin on the hourly images for all images in the continuous imagery recording (n = 536). Subsequently, these coordinates were grouped into bins, each bin covering ∼1.125 cm^2^, and maps featuring spatial presence of fauna were produced. An index quantifying the measure of spatial segregation was calculated for each species and a pairwise segregation index was calculated between neighbouring species (Dixon package in R [25]).

### 4. Statistical Analyses

Various frequencies were investigated to study faunal variations, going from hourly, every 4 hours, every 6 hours to every 12 hours. Since the probes that recorded the environmental variables were not positioned on the filmed assemblage itself, timestamps were relative and could not be considered representative; hence, the values of the environmental variables used as explanatory variables for faunal variations over time were averaged per hour for each probe separately. Only the last two Fe measurement cycles were used to calculate Fe concentrations and these were averaged per recording time (be it twice or once a day). Gaps in the recorded variables data series were a restricting and determining factor in the selection of analyses.

Whittaker-Robinson (WR) periodograms, programmed in R by P. Legendre [26], were used to unravel periods in environmental variables and faunal densities. During periodogram analysis, data time series are folded into Buys-Ballot tables with periods of 2 to a maximum of *n*/2 observations. The number of columns in the table can be restricted to a range of periods assumed to be of interest for the study [27]. The underlying statistic, measuring the amplitude, used in this periodogram function is the standard deviation of the means of the columns of the Buys-Ballot table [27]; it is plotted in the resulting graph as a function of the periods. The WR periodogram can handle missing values in the datasets, when filled in with NA values (“Not Available”). Prior to periodogram analyses, datasets were tested for stationarity. In case of lack of stationarity, trends were removed by linear regression and residuals were used in the periodogram analysis. Some degree of caution is needed during interpretation as this kind of periodogram also finds the harmonics of basic periods to be significant. Periodogram analyses were carried out for both temperature data and faunal densities using the hourly data for 23 days, with a maximum period of *n/2* (∼279.5 h).

Correlations between environmental variables and faunal densities (hourly resolution) were calculated using two-tailed correlations with permutations tests (n = 999), which is a correlation test that does not require normality of the data. P-values were subject to a Holm correction for multiple comparisons. Additional correlations using different frequencies (every 4 hours, every 6 hours and every 12 hours) were computed among faunal taxa.

Variation partitioning, by multiple regression and partial canonical analyses [28], [29], [30], was carried out on the faunal densities (excluding the visiting fish species), using two types (subsets) of explanatory variables: (1) significant temporal eigenfunctions (2) a selection of temperature probe measurements, retained in both cases by forward selection on the faunal density dataset. This analysis allowed a quantification of the proportion of variance in faunal densities explained by each subset of explanatory variables, while controlling for the effect of the other, as well as an estimation of the variation explained jointly by the two subsets.

## Results

### 1. Data Collected & Used

Total deployment time of the TEMPO-mini module amounted to ∼9 months, from 2011-09-29 to 2012-06-20. All instruments, except for the CHEMINI Fe analyser (Fe measurements came to a complete stop on 2012-03-26), kept on recording until recovery. However, all time series contained gaps, due to black-outs of the NEPTUNE observatory (sometimes adding up to a couple of days for all instrument recordings) or localised failed recordings (instrument-dependent).

**Figure 4:**
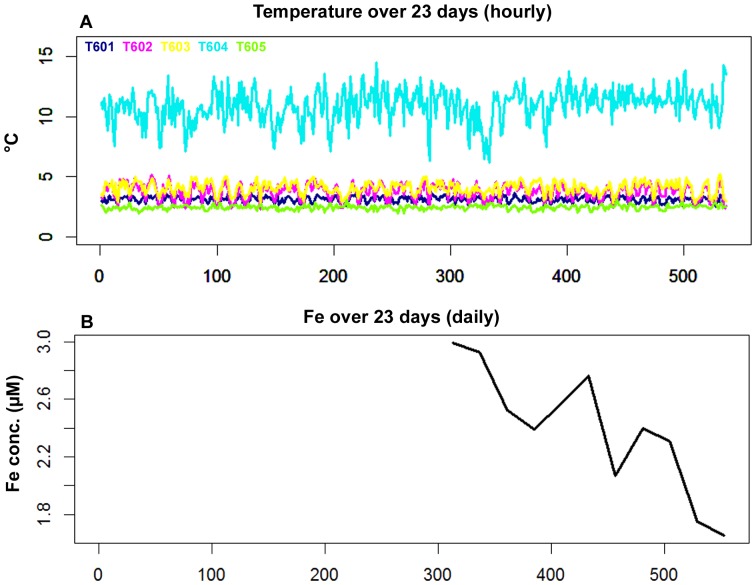
Overview of the environmental variables measured during the continous imagery recording. Overview of environmental variables measured in the vicinity of the filmed assemblage. Recording period represented here corresponds to the imagery analysis, i.e. 2011-10-07 at 8 h to 2011-20-30 at 14 h, for (A) Temperature (B) Fe concentrations. Gaps in the data during this period were due to instrument-dependent failed recordings.

From 2011-10-07 at 8 h UTC to 2011-10-30 at 14 h UTC, ∼550 hours of non-stop video were recorded and analysed with an hourly frequency, featuring 23 gaps (Fig. 3). For this period, temperature was recorded continuously (Fig. 4a), i.e. no gaps in the data. Fe measurements contained many gaps (Fig. 4b). The recording frequency of Fe measurements was changed on 2011-10-20 from three measuring cycles every twelve hours (twice a day) to once a day (at 6 h UTC), because of the drastic decrease observed in the reagents. For the oxygen measurements, a steep unexplainable decrease was noticed right after deployment; therefore oxygen measurements were omitted from the analyses of the continuous imagery recording (Fig. 3). The subsequent ∼6.5 months of oxygen data showed normalised patterns, but were outside the scope of this study.

During the non-stop imagery recording period, changes in luminosity were evident. These were assessed using a HSL index on the images, combining hue, saturation, and light (Fig. S1). A first decrease was noticeable from hours 1 to 77 (corresponding to the period from 2011-10-07 to 2011-10-10), after which several adjustments on the camera’s configuration were carried out (white balance and exposure settings) to compensate for this decrease in luminosity. Later on, similar smaller corrections were made during recordings (e.g. at about 160 h (2011-10-13 at 23 h UTC), Fig. S1). A continuous opacification of the LED lights’ lenses caused the diminution of the amount of light emitted and consequently captured by the camera. This resulted in a blackening of the recorded imagery, which rendered the images unusable after December 2011.

### 2. Community Dynamics

#### 2.1. Faunal variations and biological interactions

Six taxa were recognised on imagery and quantitatively assessed, i.e. *Ridgeia piscesae* tubeworms (Siboglinidae, Polychaeta), Polynoidae (Polychaeta), Pycnogonida (Arthropoda), Buccinidae (Mollusca), Zoarcidae (Chordata) and Majidae (Arthropoda). Their variations and observed interactions are presented in this section. For some taxa, identification was possible to the species level, for others, identification on imagery was not possible due to small sizes and/or the presence of more than one species in the area (more detailed information can be found in the Discussion section). Heat maps were created to show the distribution patterns of the mobile organisms, i.e. polynoids, pycnogonids and buccinids. Other gastropod species (limpets and snails) were visible on the imagery, though they were impossible to quantify accurately, and are only mentioned briefly. Results on the relationships between fauna and environmental variables are presented in 2.2.

**Figure 5:**
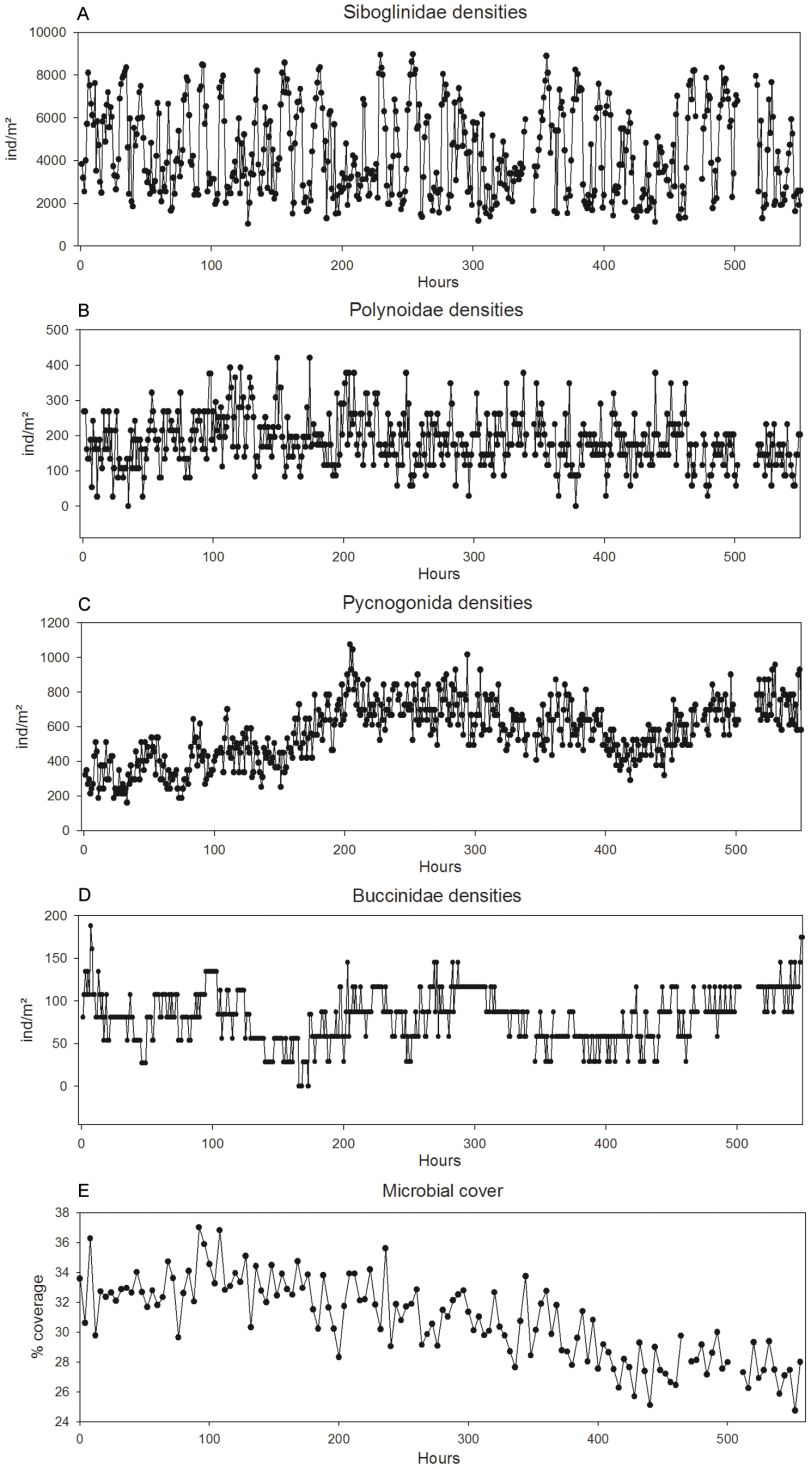
Hourly faunal densities for the continuous imagery period. Faunal densities (ind/m^2^) as assessed through image analyses with an hourly frequency. The microbial coverage (%) was measured with a 4 hour frequency. All data span from 2011-10-07 at 8 h UTC to 2011-10-30 at 14 h UTC or 559 hourly observations with some missing values (n = 23).

Siboglinidae - *Ridgeia piscesae* siboglinid tubeworms were the prime constituent and most abundant species of the filmed assemblage. Tubeworm individuals were slender, narrow and more rusty-orange in colour compared to the white *Ridgeia piscesae* tubes observed in the nearby high-flow areas. The tubeworms showed elevated activity through extension-retraction movements of their branchial plumes extending outside the tube or being completely retracted sometimes until several centimetres inside the tube. Visible *R. piscesae* densities, i.e. individuals outside their tubes, ranged from 1038 to 8980 ind/m^2^ (Fig. 5a) adding up to 8.5–78.6% of the total tubes that constituted the filmed tubeworm bush. 40.9% of the images analysed featured visible *Ridgeia piscesae* densities between 1500 and 3500 ind/m^2^. The other 47.4% was almost equally divided among the 5500–7500 ind/m^2^ (24.3%) and the 3500–5500 ind/m^2^ (23.1%) categories, while the remaining 11.7% fell in the categories >7500 ind/m^2^ (9.1%) and <1500 ind/m^2^ (2.6%) (Fig. S2a). Some of the retraction movements observed were caused by other organisms, mainly by polynoid polychaetes. Polynoid movements or actions causing retraction in tubeworm plumes were threefold, with individuals either passing over and in-between the tubeworms, scanning the tube exit with their antennae (though in a number of cases the former two types of polynoid behaviour did not provoke any reaction in the tubeworms) or by attacking the exposed tubeworm plume with their proboscis, causing them to retract into their tubes (Video S1).

**Figure 6:**
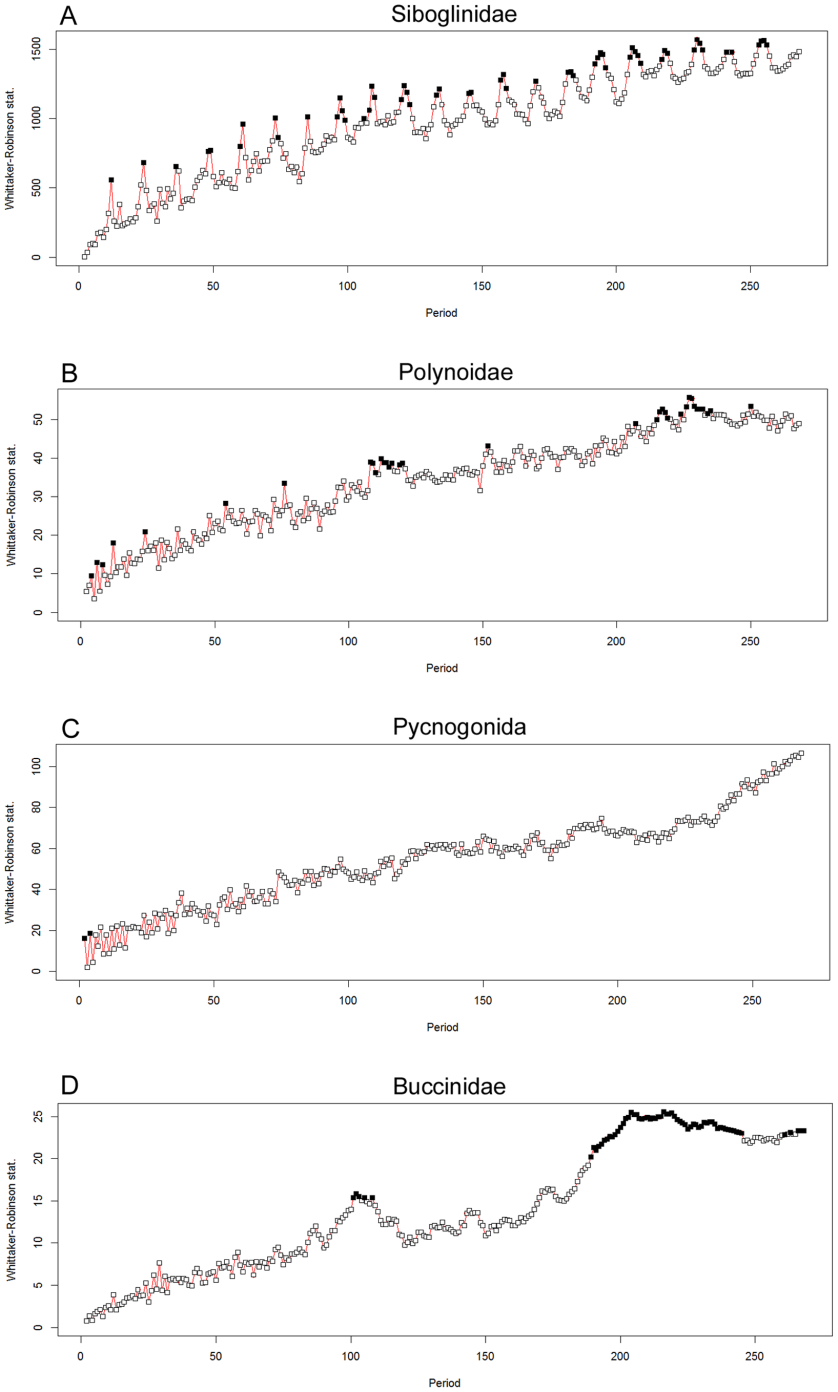
Whittacker-Robinson periodograms for faunal densities. Whittacker-Robinson periodograms computed for faunal densities, with periods spanning from 2 to *n*/2. Length of the series *n*: 559 hourly observations with some missing values (Fig. 5). Abscissa: periods in hours. The Whittaker-Robinson statistic on the ordinate is the amplitude of variation in the Buys-Ballot table, measured as the standard deviation of the table column means of the table. Black squares are significant periods for p<0.05.

A Whittaker-Robinson periodogram computed for the siboglinid tubeworm densities revealed significant periods at 12 and 24 h as well as their harmonics at 36 and 48 h. The harmonics were neatly recognisable all along the periodogram (Fig. 6a).

Polynoidae. Polynoid polychaetes of different sizes were present on the tubeworm assemblage featuring differences in number of elytra and in colour. Several individuals had elytra missing and there were three sightings of a large polynoid with its elytra completely covered by white microbial filaments. Polynoids and pycnogonids did not interfere a lot with one another, nor did polynoids and buccinids. Short of two encounters between a zoarcid fish and a polynoid individual, in which the latter swam rapidly away after a possible contact with the fishes’ head, no negative interactions of other species on polynoid presence were observed. As described in detail above, larger-sized polynoids, ca. 2–2.5 cm were occasionally observed to attack the *Ridgeia piscesae* tubeworm plumes that extended outside their tubes. Densities ranged from a minimum of 0 ind/m^2^ to a maximum of 421 ind/m^2^ (Fig. 5b), with a predominance of 70.2% of the images analysed featuring polynoid densities between 100–250 ind/m^2^ (i.e. between 4–9 individuals in the field of view) (Fig. S2b).

A Whittaker-Robinson periodogram, for periods from 2 to *n/2* (Fig. 6b), revealed significant periods at 4, 8, 12 and 24 h. Additional significant periods at 54 and 76 h were revealed, followed by a period corresponding to ∼4.5 days (108–110 h, 111–116 h and 119–120 h), 152 h and ∼9 days (207 h, 215–219 h, 224 h, 226–235 h and 250 h).

Pycnogonida - Pycnogonid densities were most likely to be an underestimation as individuals tended to cluster in crowded areas thus complicating counts especially as “stacking” was observed, i.e. various individuals were positioned on top of each other. Minimum and maximum densities observed amounted to 161 and 1075 ind/m^2^ respectively (Fig. 5c), while 25.6% of the images showed densities between 600 and 700 ind/m^2^, followed by 19.6% between 401–500 ind/m^2^ and 16.4% between 501 and 600 ind/m^2^ (Fig. S2c). No predatory behaviour was observed in the pycnogonids, nor were there any noteworthy negative interactions between the sea spiders and other taxa. A set of characteristic movements were observed of individuals dancing/bouncing up and down, bending their legs, sometimes on top of another individual.

A Whittaker-Robinson periodogram showed no significant periods except for the 2 and 4 h periods, but none of their harmonics or other periods were significant (Fig. 6c).

Buccinidae - Buccinid whelks of the species *Buccinum thermophilum* were observed moving up and around the tubeworms bush. Often these individuals had multiple limpets attached to their shells. Minimum buccinid densities ranged from absent (0 ind/m^2^) to a maximum of 188 ind/m^2^, which corresponded to 0 to 7 individuals in the field of view (Fig. 5d). 60.5% of the images analysed featured densities between 51–100 ind/m^2^ (or 2 to 4 individuals in the field of view), followed by 28.5% between 101–150 ind/m^2^ (Fig. S2d). Active feeding of buccinids was not observed, neither on *Ridgeia piscesae* individuals nor on any other visible invertebrates. Instead, they were observed scanning the environment using their extended siphons. Sometimes these gastropods tended to bury themselves into the tubeworm bush and became almost invisible to the observer’s eyes. Occasionally two individuals were positioned one on top of the other, moving jointly; sometimes the top one was “catapulted” away, rolling over and landing several cm’s below. Paired alignment between two individuals which could allow copulation was observed, but no egg masses were found in the field of view. Even though buccinids were observed to move rather swiftly, the tubeworm bush did not appear to be an ideal surface for their locomotion, as they did not appear to travel efficiently and their locomotion was more like waggling.

A Whittaker-Robinson periodogram revealed significant periods corresponding to a ∼4.5 day period (101–103, 105 and 108 h) and a large number of periods covering a ∼9 day period (189–245 h) (Fig. 6d). Additional significant periods were found at 261, 263 and 266–268 h.

Other gastropods - Lepetodrilid limpets were observed moving about, forming strands of stacked individuals, sometimes sweeping over the assemblage. However due to the zoom level and their high abundances, no accurate quantification was possible. Provannid snails were also recognisable on some of the zoomed-in sequences (before 2011-10-07) but only seldom on the continuous recordings. Their small size and fairly dark colour made individuals hard to discern.

Zoarcidae - At most two individuals of zoarcid fish were sporadically encountered on the video imagery. Due to their low abundances and many zeros, they were not included in the statistical analyses, but their behaviour and activity was noted. Zoarcid individuals tended to position themselves most often on the tubeworm bush (20 observations or 3.73% of the hourly images analysed) often followed by hiding in-between the tubeworms or underneath the bush. Sometimes they were seen next to the bush (12 observations = 2.23% of images analysed). Two cases of possibly negative interactions were observed, involving a polynoid polychaete. The latter swam swiftly away after a possible contact with a fishes’ head; this kind of fleeing behaviour appeared in both cases to be induced by the zoarcid fish. This fish species could have been impacted by the lights of the TEMPO-mini module, since they were always present in the field of view after a black-out.

Majidae - A majid spider crab visited the tubeworm bush several times. Eleven observations were made on the imagery, some of them only separated by a couple of minutes. A first visit took place on day 13, on 2011-10-20 at 7∶59 h UTC, followed by several subsequent visits (n>5, last visit on 2011-10-27, 16 h UTC (day 20)). The nature of these visits consisted out of the spider crab “touring” the assemblage, often placing its legs straight into the faunal assemblage. These actions did not prompt any reactions in the other taxa. Even when the majid crab placed one leg right next to a buccinid snail it did not trigger any reaction. During one of its other visits, the spider crab sat on the camera module with its legs dangling in front of the lens, thus putting the assemblage out of focus. Zooming in on the spider crabs’ legs, two amphipods, of the Caprellidae family were recognised (Figure S3).

Microbial cover - The percentage (%) microbial cover, quantified every 4 hours, showed a clear decrease over time (Fig. 5e). A significant negative correlation was found with the pycnogonid densities (r = 0.30, p = 0.016, for 133 observations (n)).

**Figure 7:**
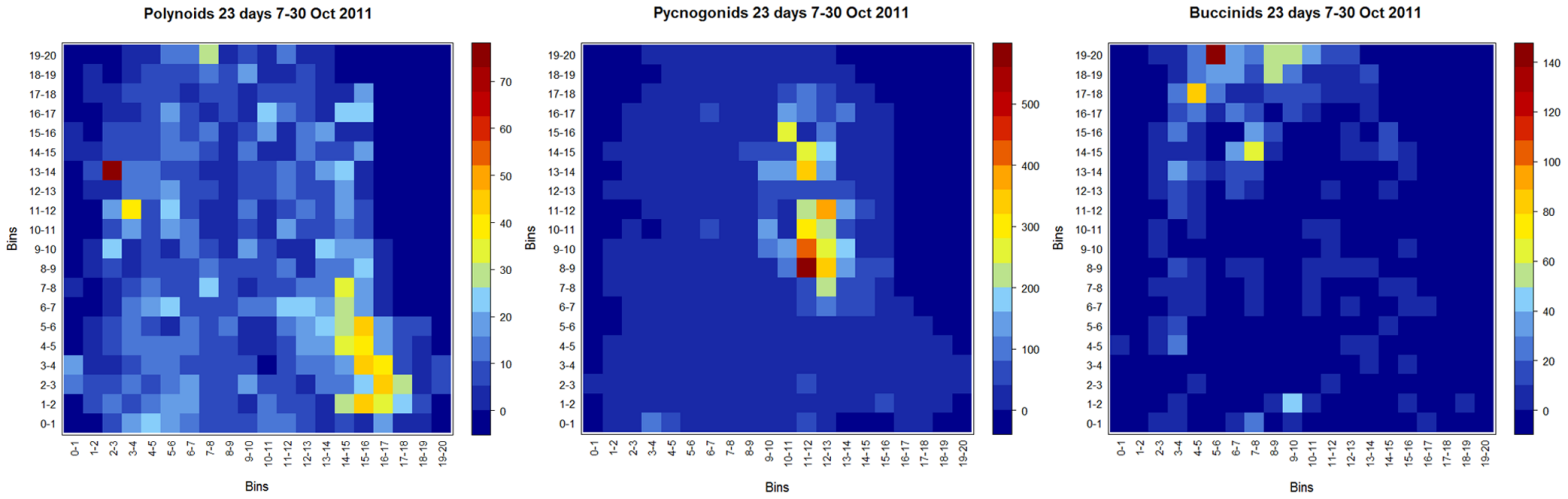
Heat maps of polynoids, pycnogonids and buccinids. Heat maps showing the distribution of the three mobile species on the filmed tubeworm bush, based on the number of observations during the 559-coded per bin (1 bin = ∼1.125 cm^2^). Colour-coded legends along with the corresponding number of observations can be found next to each plot.

Faunal relations - Following the polynoid attacks on the *Ridgeia piscesae* plumes, there was a significant negative correlation between the densities of tubeworms out of their tubes and polynoids (r = −0.349, p = 0.012, n = 536), and that for all frequencies studied. The relationship between pycnogonids and tubeworms was characterised by a negative correlation that was not significant for the hourly frequency (r = −0.089, p = 0.28, n = 536), nor for the other frequencies where it sometimes inverts (4 h: r = −0.012, p = 1.00, n = 133; 6 h: r = 0.091, p = 1.00, n = 90; 12 h: r = 0.33, p = 0.42, n = 45). There appeared to be little interference or interactions among polynoids, pycnogonids and buccinids. This was corroborated by heat maps, in which there was a clear distinction between the distributions of these three mobile taxa (Fig. 7). Spatial segregation tests were carried out on the point locations used in the heat maps for the three taxa by analysing the counts in a nearest neighbour contingency table. For all taxa, the observed counts of conspecific neighbours were larger than the expected counts, meaning that each taxon was associated with their conspecific neighbours (Table 1). For the polynoids, the measure of segregation (S [31]) also confirmed that their nearest neighbour was less likely to be a pycnogonid or a buccinid (negative S-values) and thus more likely to be another polynoid (positive S-values) (Table 1). The tendency to segregate was high in the polynoids (high S-values, Table 1), which was confirmed by looking at the heat maps where polynoids tended to move around a lot, covering the entire tubeworm bush (Fig. 7). The lack of distinct interactions between polynoids and pycnogonids was also reflected in the correlations, as for the hourly, 4 and 6-hourly frequencies no significant relations between the two taxa were revealed (p>0.4), exception being the significant negative relation at the 12 h frequency (r = −0.39, p = 0.036, n = 45). Polynoids and buccinids, on the other hand, showed no significant correlations, regardless of the frequency studied (p ∼1.00). The clustering behaviour in pycnogonids was illustrated by the heat maps (Fig. 7) and spatial segregation tests, showing the lowest tendency to segregate (Table 1). A significant positive relation was revealed on an hourly frequency for the pycnogonids and the buccinids (r = 0.16, p = 0.02, n = 536), but not for the other frequencies (p∼1.00). Of all taxa, buccinids had the highest tendency to segregate (Table 1), though the surface area covered was not as widespread as observed for polynoids.

**Table 1:** Measures of spatial segregation [31] between three mobile taxa and neighbours based on all hourly observations during the 23 days continuous recording period (n = 559–23 missing).

From	To (neighbour)	Obs. Count	Exp. Count	S
Buccinidae	Buccinidae	**1024**	142.86	1.31
	Polynoidae	186	330.20	−0.30
	Pycnogonida	284	1020.94	−0.96
Polynoidae	Buccinidae	206	330.20	−0.22
	Polynoidae	**1750**	762.52	0.56
	Pycnogonida	1495	2358.28	−0.45
Pycnogonida	Buccinidae	323	1020.94	−0.53
	Polynoidae	1633	2358.28	−0.20
	Pycnogonida	**8714**	7290.78	0.31

The observed values (Obs. Count) larger than the corresponding expected values (Exp. Count) are in bold. Values of S calculated for a taxon and its conspecific neighbour larger than 0 indicate that the taxon is segregated; the larger the value of S, the more pronounced the segregation. These values of S (between conspecific neighbours) closer to 0 are consistent with random labelling of the neighbours of species. The S between two different taxa is less than 0 because the observed frequency of a taxon as a neighbour is smaller than expected under random labelling.

#### 2.2. Links with environment

Environmental variable time series started recording on 2011-09-29, though the results discussed here were selected to correspond to the continuous recording period, starting 2011-10-07 (Fig. 4). The correlations between fauna and environment presented here should be considered approximate, as the environmental variables were not recorded directly on the filmed assemblage itself (Fig. 2). More detailed information on this can be found in the Discussion section.

Temperature - Temperature values during the continuous imagery recordings for this study varied between 1.92°C and 14.47°C, with means ranging from 2.47 (T605) to 10.89°C (T604), though predominantly around 3.08°C to 4.06°C (T601, T602 and T603). For the 5 deployed temperature probes (Fig. 2), T604 showed the highest temperature values measured as well as the largest variations (Table 2), whereas the T605 probe recorded the lowest values, approaching the surrounding seawater temperature (∼1.8°C). Significant correlations among the five temperature probes and the faunal variations were observed. Most significant were the positive correlations between T602 and T603 with the visible tubeworm densities (r = 0.49 and r = 0.62 respectively, p = 0.02, n = 536) and a significant negative one with T605 (r = −0.30, p = 0.02, n = 536). Significant negative correlations were observed between the same two temperature probes (T602 and T603) and polynoid densities (r = −0.17 and r = −0.23 respectively, p = 0.02, n = 536).

**Table 2:** Overview of the environmental data recorded in the vicinity of the TEMPO-mini ecological module at the Grotto hydrothermal vent.

	23 days continuous imagery				
	min	max	mean	stdev	var
**Temperature (°C)**					
T601	2.45	3.68	3.08	0.27	0.08
T602	2.28	5.14	3.76	0.65	0.42
T603	2.73	5.27	4.06	0.51	0.26
T604	**6.19**	**14.47**	**10.89**	**1.37**	**1.87**
T605	1.92	3.12	2.47	0.18	0.03
**Fe (µM)**	1.65	2.99	2.39	0.44	0.19

Highest values for temperature were in bold. Min: minimum, max: maximum, stdev: standard deviation, var: variance.

Fe - The minimum Fe concentrations ranged from 1.65 to a maximum of 2.99 µM during the continuous imagery recordings, while the mean was 2.39±0.44 µM (Table 2). No significant relationships were revealed between the faunal variations and the Fe measurements available in the period October 20–30, 2011, featuring a resolution of one measurement a day (24 h frequency).

**Figure 8:**
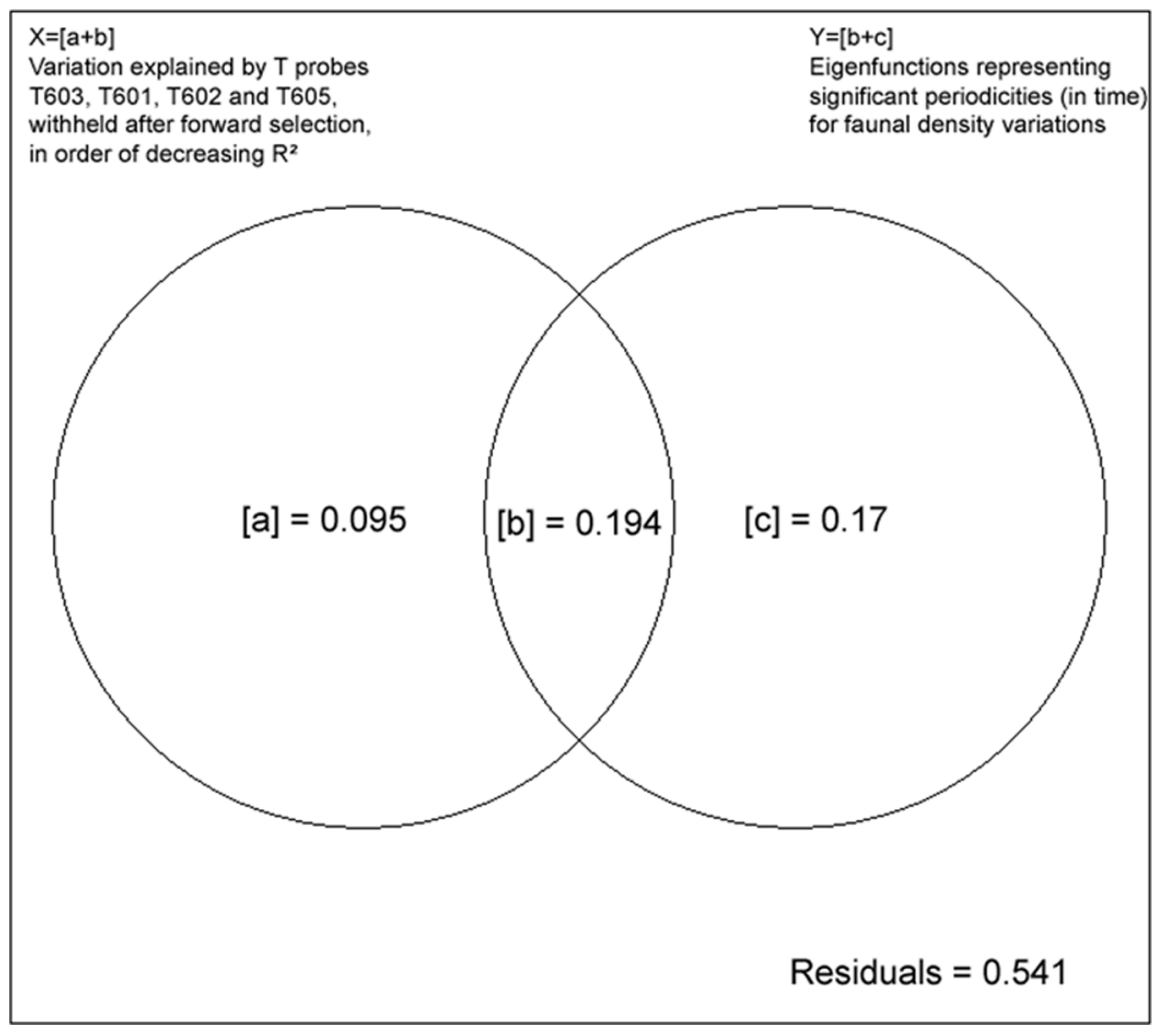
Variation partitioning for the faunal density data. Variation partitioning for the faunal density data (excluding the zoarcid fish densities), using two types of explanatory variables: X = a selection of different temperature probes, and Y = significant temporal eigenfunctions, retained in both cases by forward selection on the faunal density dataset. [b] is the variation explained jointly by X and Y. The residuals represent the amount of variation not explained by a linear model of the two sets of explanatory variables.

Variation partitioning - Variation partitioning allowed quantification of the variation explained by two subsets of variables with a Venn diagram representation (Fig. 8). The significant variables retained by forward selection against the faunal variations were the temperature measurements from probes T601, T602, T603 and T605, as well as a subset of 33 significant temporal eigenfunctions. Most of the variation in faunal densities was explained by temporal periods represented by the significant eigenfunctions, adding up to 36.5% (Y = [b+c], Fig. 8). X on the other hand represented the variation explained by the subset of temperature variables (X = [a+b]); it explained 28.9% of the faunal variation. Of these percentages, 19.4% of the variation ([b]) is explained jointly by the temporal eigenfunctions and the temperature values, meaning that these variables showed collinearity (i.e. correlation). A small percentage ([a] = 9.5%) of the variation was explained solely by the temperature variables while other temporal periods found in the faunal densities were only modelled by the temporal eigenfunctions ([c] = 17.2%). All fractions were significant, with p = 0.001. The 54.1% residuals correspond to the faunal variation not explained by the temperature variables and eigenfunctions.

### 3. Temporal Variation in Environmental Conditions

#### 3.1. Temperature

**Figure 9:**
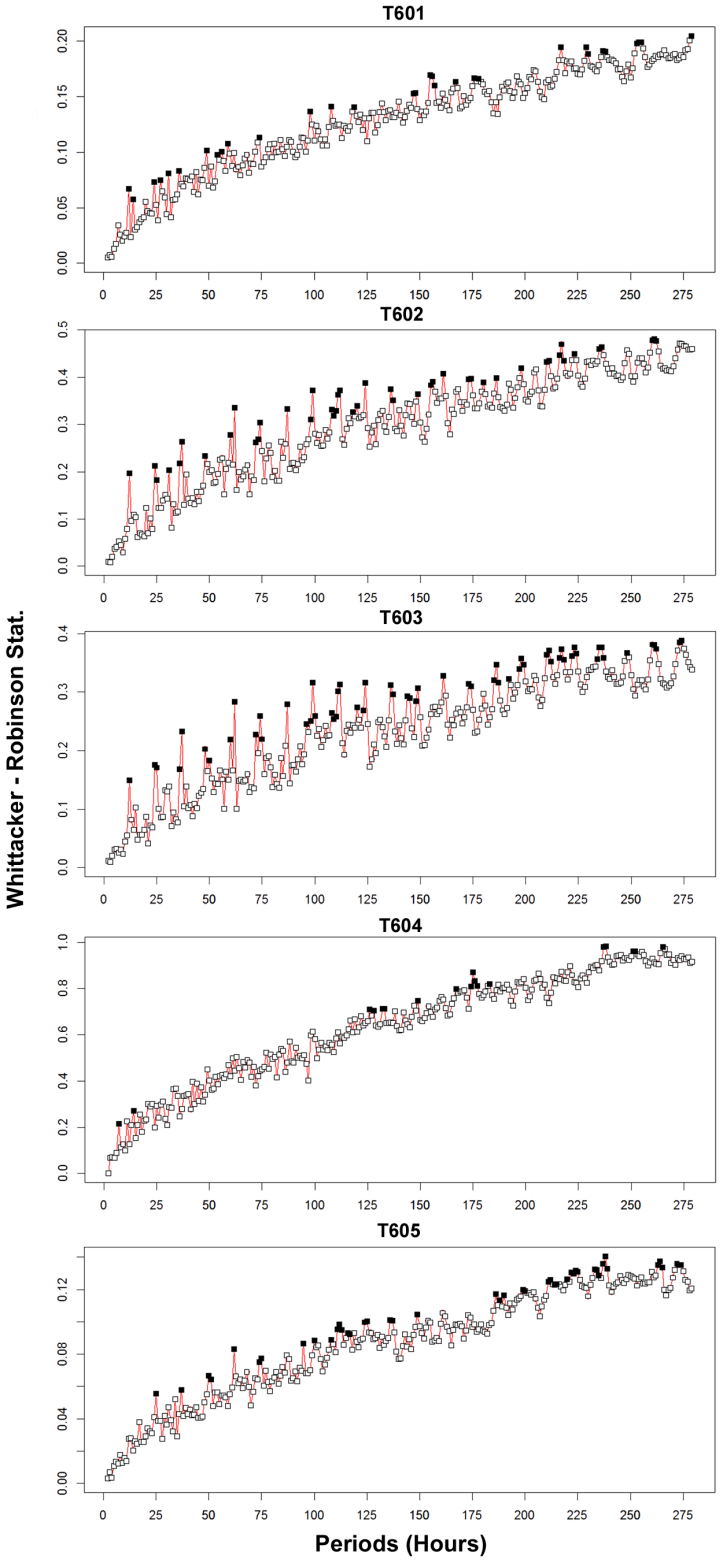
Whittaker-Robinson periodograms for 5 temperature probes. Whittaker-Robinson periodograms computed for the detrended temperature values of all five probes (locations: see Fig. 2) during the 559 h or 23 day continuous imagery period. Abscissa: periods in hours. The Whittaker-Robinson statistic on the ordinate is the amplitude of variation in the Buys-Ballot table, measured as the standard deviation of the table column means. Black squares are significant periods at p<0.05.

All 23 day temperature time series, from the different temperature probes, except T603, showed significant trends and thus did not comply with the stationarity requirement for periodic analysis. Hence, trends were removed and periodogram analysis was carried out on the residuals for periods of 2 to *n*/2 (279.5 h∼11.5 days). Diurnal and semi-diurnal periods, and their harmonics, were the main significant frequencies discerned. No clear or distinct significant multiple day cycles were encountered. Significant periods at 25 h were revealed for all but one temperature time-series, the exception being probe T604. An additional significant period at 12 h was revealed for probes T601, T602 and T603, while for all temperature probes recurrent harmonics of both semi-diurnal (12 h) and diurnal (25 h) frequencies were identifiable throughout the temperature time series, which agree well with the tidal cycle (12 h 25 min and 24 h 50 min) (Fig. 9). The periodograms of the first three probes (T601, T602 and T603) showed more similar patterns, while T604 had almost no significant periods and T605 appeared more intermediate between these two graph types.

#### 3.2. Fe

Only a short period of ten days (2011-10-20 to 2011-10-30) with one measurement per day was available for Fe time-series analyses during the continuous imagery recordings, which proved too short to reveal any significant periods by the periodogram analysis.

## Discussion

### 1. Ecological Framework of the Observatory Deployment

#### Fauna

The filmed assemblage was recognized as the low-flow variant of community V, described by [7], featuring narrow *Ridgeia piscesae* tubes and abundant gastropod fauna. On basalts, long skinny *R. piscesae* densities were shown to be able to reach densities up to 200000–300000 tubes/m^2^ [18], while sampling of a similar assemblage on a sulphide structure revealed densities up to 66903 ind/m^2^ [17]; both are much higher than the visual counts made during this study (<9000 ind/m^2^). While gastropod species were very abundant in this type of assemblage [7], [17], our image analysis study did not really reflect that, as only buccinids were large enough to be quantified on the imagery. High abundances of lepetodrilid individuals (*Lepetodrilus fucensis*) were detected and occasionally, a third gastropod species, *Provanna variabilis,* was visible (unpublished results, ground-truth sampling during ‘Wiring the abyss cruise 2013’ (June 2013)), but both were not quantifiable based on imagery. Community V low-flow was also characterised by high abundances of pycnogonids and polynoids, which were shown to be more numerous than in the other assemblages at Endeavour vents [17]. Pycnogonids were very abundant on the footage analysed here, with higher densities (ind/m^2^) than reported previously [17], even though our values still represent an underestimation due to stacking. The ground-truth sampling at Grotto - of an assemblage similar to the one filmed - also revealed the presence of *Paralvinella* cf. *palmiformis* (unpublished results, ‘Wiring the abyss cruise 2013’), which were unaccounted for on imagery but were present in the community V low-flow as described by [17].

#### Environmental variables

The positioning of the probes recording environmental variables was not optimal for the purposes and objectives of studying the community dynamics of the deep-sea hydrothermal vent fauna at MEF and linking it to its environment. The probes were deployed rather hastily because of a storm threat at the surface, cutting short the diving time. Nevertheless, following [7], T602 and T603 featured an assemblage corresponding to community IV. This specific community (IV), characterised by limpets, snails and small *R. piscesae* [7], was indicated as the one preceding the filmed assemblage (i.e. community V low-flow) in the succession model proposed by Sarrazin et al. [7], and as such the most similar to the filmed assemblage regarding the environmental conditions. For the other probes, T601 appeared to be positioned on a similar assemblage as T602 and T603, although it was not really in touch with the assemblage in question. T604 featured the warmest temperatures on an assemblage recognisable by numerical abundant white limpets and *Paralvinella* palm worms, corresponding to the warmer community III [7]. Dense clouds of shimmering water bathed this community in warm fluids. Contrastingly, the T605 probe approached the ambient seawater temperature, though sometimes the influence of warmer fluids, through rising shimmering water plumes, was detectable. For the CHEMINI probe, there was some doubt that this sensor/probe was actually in contact with the fauna. It was deployed on a similar assemblage as the one filmed. The fact that the sampling inlet was not in touch with the faunal assemblage, along with the distance separating it from the field of view, might (at least partially) explain the lack of correlation/relationships with the faunal variations.

### 2. Temporal Community Dynamics

Temporal community dynamics between the taxonomic groups quantitatively assessed on imagery revealed insights in their daily interactions and mutual relationships. Depending on the frequency studied (hourly, 4 h, 6 h or 12 h), the correlations between faunal taxa might change, from statistically significant to not significant or sometimes even inverting the relationship.

#### Siboglinidae

Of all vestimeniferans, *Ridgeia piscesae* are known to exhibit greater tolerance to varying physicochemical conditions [32]. This was also confirmed by the different morphologies (ecotypes) of the species where a low-flow variant was characterised by skinny vestimentifera with poorly developed branchial filaments while high-flow vestimentifera had short wide white tubes and prominent, feathery branchial plumes [17], [33]. Even with poorly developed branchial plumes, the filmed rusty-coloured skinny *Ridgeia* individuals showed quite some extension-retraction movements. While several plume retractions of *R. piscesae* were caused by polynoid attacks, in the majority of cases no visible external trigger could be identified. The extension/retraction movements of *R. piscesae* were following what appeared to be an individual rhythm. Nevertheless, this observatory approach allowed us to identify distinct semi-diurnal and diurnal rhythms in emergence/retraction of tubeworm plumes for the filmed assemblage as a whole, which were recognisable throughout the entire time series. Currents, fluid flow, temperature and oxygen have all been hypothesised to play a role in the occurrence of these rhythms in tubeworms. However, no consistent statistically significant patterns linking tubeworm (*R. piscesae*) extension/retraction to high currents, changes in currents or turbidity were found [10]. In the present study, correlations with temperature were revealed, although these should be treated with some caution due to the sub-optimal probe deployment. Emergence/retraction movements of siboglinid tubeworms were also proposed to be a thermoregulatory behaviour or suggested to be governed by oxygen requirements [10], [34]. Urcuyo et al. [18] indicated that most of the time individuals in a long-skinny tubeworm assemblage were exposed to extremely low levels of vent fluid and sulphide while their posterior sections were consistently immersed in sulphide concentrations 1000 times higher, which led these authors to suggest that this long-skinny ecotype might have the capacity/ability to acquire sulphide through its posterior end (root-like structure). If this is taken into account in the interpretation of our results, the extension/retraction movements could indeed be a consequence of changing sulphide and oxygen saturation levels/requirements, along with an associated regulatory behaviour, i.e. through increasing or decreasing the distance between the source of the higher temperature and sulphide concentrations.

No reproductive ‘actions’ were observed during this study. While free spawning events have been observed for *Riftia pachyptila* [35], *Ridgeia piscesae* tubeworms transfer sperm packages (tiny white thread-like objects) from male to female using the branchial filaments of the branchial plumes [36]. The tiny size of the sperm packages, the poorly developed branchial plumes [36] of the studied assemblage as well as the relative short time span of the images analysed could explain the lack of such observations for the studied assemblage.

#### Polynoidae

Different species of polynoids were observed on the imagery, characterised by differences in sizes, colours and number of elytra. Polynoids known to inhabit MEF vents are *Lepidonotopodium piscesae* and *Branchinotogluma* sp. [19]. Four *Branchinotogluma* species were initially recognised on the Juan de Fuca Ridge: *B. hessleri, B. grasslei*, *B. sandersi* and *Opisthotrochopodus tunnicliffeae*, but the last three have recently been treated as one single species, i.e. *Branchinotogluma tunnicliffae* [37], [38]. Even though it cannot be used as a distinguishing species characteristic, *L. piscesae* has been observed with bacterial-coating [7], [19], [37]. Several polynoids had scales missing, which could be evidence of predation or intraspecific fights [39], though no such behaviour was observed on the TEMPO-mini video footage. Additionally, their movements in between the tubeworms might prevent the elytra from regenerating (the loss of elytra in scale worms was hypothesised as being an adaptation to an interstitial environment [40]). Based on the heat maps and individual recognisability, certain polynoids were observed to appear and dwell in the same region, which raised the question of territoriality or homing patterns. Differences in local physico-chemical conditions on the tubeworm bush and microhabitat preference could contribute to this pattern.

#### Pycnogonida

At least one pycnogonid species, *Ammothea verenae*, is known from the neighbouring vents at MEF (known from Bastille edifice [41] and from scenescent/inactive samples at Endeavour [42]). Hence, it is likely that this species also inhabits the Grotto hydrothermal vent. It was reassigned to the genus of *Sericosura* by Bamber [43]. One of the noteworthy behaviours observed were the pycnogonids bouncing up and down on top of each other. This kind of ‘dancing’ or ‘pumping’ behaviour in females is recognised as (an initiation to) mating behaviour [44]. No negative interactions were observed between pycnogonids and tubeworms on the video imagery, more precisely, pycnogonids did not invariably cause retraction of tubeworm plumes, nor were they observed to attack or nibble on tubeworm individuals. Therefore their presence and abundance appeared more likely linked to different environmental conditions in which they tended to prevail. This was confirmed by pycnogonids showing opposite correlations with environmental variables when compared to tubeworms. Moreover, the pycnogonids were also gregarious in a very localised region of the field of view. Territoriality and suitable microhabitat could thus both explain this observed distribution pattern. There was also a significant negative correlation between microbial cover and pycnogonid densities. As *Sericosura (Ammothea) verenae* was shown to be a part of the bacterivore feeding guild [45] it could, through increasing densities and feeding (grazing), decrease the percentage microbial cover. No recurrent rhythms in pycnogonid abundance or temporal variation could be identified. Its quantification was most prone to sampling bias and possible observer effects/experience due to stacking of individuals; therefore densities presented here should be considered more indicative than quantitative.

#### Buccinidae

The buccinid gastropod species *Buccinum thermophilum* was also identified from other edifices of the MEF neighbouring Grotto [46]. Analysis of stomach contents of this species in a previous study included basalt chips, vestimentiferan tube parts, pycnogonid legs, polychaete setae and unidentified flesh [46]. We were unable to confirm that these whelks indeed fed on *Ridgeia piscesae* or on pycnogonids or polychaetes, as no such feeding actions were observed. The snails were seen burying themselves in the tubeworm bush, where they could possibly be feeding on smaller fauna (e.g. small crustaceans) living in interstitial spaces. This was also put forward by Martell et al. [46], who stated that, based on the lack of substratum preference, *B. thermophilum* were more likely to feed on small mobile prey rather than on vestimentiferans. The upward extended siphons in this species, observed in the present study, differed from its littoral conspecific, and could represent a response against rising sulphide-rich plumes [46]. This species featured the highest spatial segregation, not due to the large area covered, but rather because of their low densities, i.e. not much possibility to have or find conspecific neighbours. Significant periods were revealed at 4.5 days and ∼9 days, though tidal-related (semi-diurnal) signals were not significant. For comparison, during a 62-day continuous video recording in a Sagami Bay cold seep at 1100 m depth, no infradian rhythms were found in visual *Buccinum* snail counts, though a significant semi-diurnal tidal component (12 h) was discernable [47].

#### Zoarcidae

Observed eelpout fishes of the Zoarcidae family could belong to the genus *Pachycara*, which has been reported from Endeavour. These eelpouts exhibited swift movements, which supposedly could represent feeding actions; however no specific impact was made out on the hydrothermal fauna in the field of view. There were at most 2 individuals present in the field of view and, based on their presence/absence patterns, an effect of lighting was assumed. The effect of lights was suggested to be stronger for eelpouts when compared to invertebrates [47].

#### Majidae

A majid spider crab, most likely *Macroregonia macrochira,* known from submersible and towed camera photographs from the Juan de Fuca and Explorer ridges and in high concentrations from around hydrothermal vents [48], visited on several accounts the filmed assemblage. Despite being known as a major predator of hydrothermal vent animals [2], [18] and more specifically of *Buccinum thermophilum* snails [46], it did not cause fleeing reactions in the mobile fauna or retractions in tubeworms during its visits, nor were there any predation activities observed. Caprellid amphipods, as seen attached on the spider crab’s legs, were previously identified as a new species attached to the *M. macrochira* spider crab’s mouthparts and constituted the first record of this family close to hydrothermal vents (*Caprella bathytatos* from Endeavour vents [49]).

#### Effects of lighting

Lights were continuously powered on during the experimentation presented here. Whether this impacted the studied fauna is hard to discern, except for the apparent impact on fish presence. We observed no distinct decline in faunal invertebrate abundances linked with the duration of the experiment and consequently the exposure to continuous light.

#### Links with environmental variables

The distinct differences in spatial distribution for the three mobile taxa (polynoids, pycnogonids and buccinids) observed in this study could reflect different microhabitats, which could be corroborated by the different correlations between the faunal densities and temperature measurements from the different probes. Overall links between the community dynamics and the environmental variables were visualised with the variation partitioning. Most variation explained was shared between temporal periods and temperature, followed by the variation explained solely by the temporal periods. Additionally, only a very small fraction was explained exclusively by the temperature measured. This thus implies that there were temporal periods explaining variation in faunal densities that were not recognised in the environmental variables measured as such. The explanation for this can be three-fold, with the explanations not being mutually exclusive and more likely to be entwined. For one, this pattern could at least partially be attributed to the positioning of the thermistor array, which was deployed in proximity of the filmed assemblage instead of on it. Hydrothermal vents are characterised by steep gradients in environmental variables and a high local variability on a scale of centimetres [50], [51], which could account for some of the discrepancies observed. The elevated spatial variation of abiotic conditions at vents was also illustrated by the differences observed between the different deployed temperature probes. Even though the probes were only separated by several decimetres at most, the same significant periods were not always as easily recognised amongst them. Local hydrodynamic conditions and bottom surface currents could influence temperature measurements on a scale of tens of centimetres [52]. Secondly, it is more than likely that there are other (non-measured) environmental factors at play, causing variations in the faunal abundances [50]. This was emphasised by the prominent amount of variation that remained unexplained. Thirdly, the organisms within the assemblage might have different individual rhythms based on energy requirement and saturation, hence revealing additional significant temporal periods. This could also be linked with the size of the individuals. Alternatively there could be a delay in reaction time between the environmental variables measured and the response of the fauna.

### 3. Temporal Variations in Environmental Variables

#### Temperature

The temperature measured in the vicinity of the filmed assemblage ranged within the limits of what is typically referred to as low-temperature fluid (5–75°C) or ‘diffuse’ hydrothermal flow [52]. In the temperature series, diurnal periods at ∼25 h were discerned. Significant semi-diurnal periods were also found, although sometimes they could only be identified based on their harmonics. These semi-diurnal and diurnal periods and harmonics were particularly clear in T601, T602 and T603, but less in the data originating from the warmer T604. Explanation for this pattern could be due to the waning influence of the tides as the heat flux strength increases [52]. The influence of currents and local hydrodynamic conditions was visible in the case of T605 as well, occasionally bathing it in warmer fluids which might have possibly masked some of the tidal periods in the surrounding seawater.

Tidal patterns in environmental variables could be attributed to a modulation of the fluid flow or the fluid flow rate. In the Barkley canyon, another instrumented node of the NEPTUNE network at 870 m depth and closer to the shore, periods of enhanced bottom currents associated with diurnal shelf waves, internal semidiurnal tides, and also wind-generated near-inertial motions were shown to modulate methane seepage [53]. Measured tides, based on pressure data recorded by a camera platform CTD in the same canyon, were mixed semidiurnal/diurnal [54]. However, temperature variability at hydrothermal vents was shown to greatly diminish when current directions did not shift in direction with the tides [55]. Hence, it was suggested that temperature variability correlates with the variability of the current speed and direction, not with the ocean tidal pressure [55]. The modulation of temperature by tides is thus only indirect, through the modulation of horizontal bottom currents by tides. A current meter on a future deployment of the TEMPO-mini module would allow to test this hypothesis and to quantify a possible impact of the currents on the fauna in more detail.

#### Fe (iron)

Fe is used as a proxy for vent fluid composition. However, no significant periods were found in the Fe measurements for the duration of the deployment. Nor were there any significant links established with the faunal variations in any of the analysed taxa. Semi-diurnal and diurnal periods could not be determined because the shortest period that can be resolved is twice the interval between the observations of the time-series (in this case 2×24 h after 2011-10-20) [27]. The ‘lower’ sampling resolution, in addition to the many gaps in the time series, might explain why no relationship was found between the faunal taxa and Fe concentrations.

## On Rhythms and Recording Frequencies and Other Conclusions

Overall, tidal rhythms were shown to be at play at 2200 m depth in the hydrothermal vent ecosystem at MEF, both in fauna and environment. For the fauna, the identified rhythms may either be controlled by an internal biological clock or constitute a response of the organism to changing environmental conditions [47]. For the neat semi-diurnal and diurnal cycles in the tubeworms’ appearance, the question thus remains whether these rhythms were induced by temperature, currents or other environmental variables (or a combination of them) or if they were driven by an endogenous individual/programmed trigger. For the other taxa (polynoids, pycnogonids and buccinids), tidal cycles could be blurred by biological forcing (e.g. predation and competition) or by higher-frequency changes in environmental conditions [56]. In our study, the environmental variables (a selection of several temperature probes) only explained about one-third of the variation encountered in the densities of the faunal assemblage over time. For future deployments, a more optimised environmental probe deployment, possibly including additional instruments (e.g. a current meter), should be conceived, taking into account the challenges of the deployment site at Grotto hydrothermal vent. This will allow for a more detailed assessment of entrainment between the rhythms in fauna and local environment.

The necessary importance should also be attributed to the programming of recording frequencies, taking into account the instrument’s limitations, as these frequencies have an impact on the resolutions of the rhythms revealed (i.e. the shortest period that can be resolved is twice the interval between the observations of the time-series) and on their use as explanatory variables. Additional caution is needed because, depending on the frequencies investigated, the type of relationships (significance, positive or negative) between the faunal taxa might be subject to change.

In summary, there were clear differences in distribution for polynoids, buccinids and pycnogonids (with the latter showing a well-defined clustering behaviour) on the tubeworm bush, which could be indicative of different microhabitats. Negative (predatory) interactions with polynoids attacking branchial tubeworm plumes were observed, while visits of large predators (Zoarcidae and Majidae) did not cause quantifiable or apparent reactions in the observed taxa of the filmed community/assemblage. A general decrease in microbial cover coincided with increasing numbers of bacterivorous pycnogonids. Alleged mating behaviour was observed in pycnogonids, though for other taxa such observations were less obvious or unobserved. Longer time series, alternated with regulated zoomed-in recordings, will grant us more thorough and additional insights.

Even though our ecological analyses were, for now, limited to an hourly resolution, we need to emphasise the importance of the higher resolution (i.e. <1 h) observation frequencies. Additionally, longer time series of faunal densities will enlighten us on the ‘representativeness’ of the multiple-day periods as found in this study and possible other infradian rhythms. The time-consuming aspect of manual imagery analysis, however, is a definite limiting factor for the feasibility of such a study. In order to achieve such a fine-scale resolution and/or longer time-series analysis, the development of automated and/or semi-automated imagery analysis tools is indispensable.

## Supporting Information

Figure S1 **HSL (hue, saturation and light) index of imagery recorded.** HSL index, combining hue, saturation and light, was calculated to assess the quality of the hourly imagery recorded during the continuous recording period (2011-10-07 to 2011-10-30). The HSL index, whose maximum value is 255 (white) and minimum is 0 (black), shows the general darkening of the recorded imagery with time.
(TIF)

Figure S2 **Frequency of faunal density observations per taxon.** Percentage of the images observed with faunal densities in defined categories. N = 559-23 gaps, i.e. 536 images analysed.
(TIF)

Figure S3 **Spider crab on top of TEMPO-mini module.** A majid spider crab, probably *Macroregonia macrochira*, sitting on top of the TEMPO-mini module. Two caprellid individuals attached to its legs can be recognised (possibly *Caprella bathytatos,* Caprellidae, Amphipoda).
(TIF)

Video S1 **A polynoid polychaete attacking a tubeworm plume.** A polynoid polychaete attacking, with its proboscis, an extended branchial plume of a Ridgeia piscesae tubeworm, causing it to retract into its tube. Footage recorded at 2186 m depth at the Grotto hydrothermal vent (Main Endeavour Field).
(WMV)

## References

[1] Cuvelier D, de Busserolles F, Lavaud R, Floc’h E, Fabri M-C, et al (2012) Biological data extraction from imagery - How far can we go? A case study from the Mid-Atlantic Ridge. Mar Env Res 82: 15–27. 23058949 10.1016/j.marenvres.2012.09.001

[2] Tunnicliffe V, Embley RW, Holden J, Butterfield DA, Massoth G, et al (1997) Biological colonization of new hydrothermal vents following an eruption on Juan de Fuca Ridge. Deep Sea Res Pt I 44(9–10): 1627–1644.

[3] Shank TM, Fornari DJ, Von Damm KL, Lilley MD, Haymon RM, et al (1998) Temporal and spatial patterns of biological community development at nascent deep-sea hydrothermal vents (9° 50N, East Pacific Rise ). Deep Sea Res Pt II 45: 465–515.

[4] Tsurumi M, Tunnicliffe V (2001) Characteristics of a hydrothermal vent assemblage on a volcanically active segment of Juan de Fuca Ridge, northeast Pacific. Can J Fish Aquat Sci 58(3): 530–542.

[5] Marcus J, Tunnicliffe V, Butterfield DA (2009) Post-eruption succession of macrofaunal communities at diffuse flow hydrothermal vents on Axial Volcano, Juan de Fuca Ridge, Northeast Pacific. Deep Sea Res Pt II 56(19–20): 1586–1598.

[6] Tunnicliffe V, Juniper SK (1990) Dynamic character of the hydrothermal vent habitat and the nature of sulphide chimney fauna. Prog Oceanogr 24(1–4): 1–13.

[7] Sarrazin J, Robigou V, Juniper S, Delaney J (1997) Biological and geological dynamics over four years on a high-temperature sulfide structure at the Juan de Fuca Ridge hydrothermal observatory. Mar Ecol Prog Ser 153: 5–24.

[8] Desbruyères D (1998) Temporal variations in the vent communities on the East Pacific Rise and Galápagos Spreading Centre: a review of present knowledge. Cah Biol Mar 39: 241–244.

[9] Cuvelier D, Sarrazin J, Colaço A, Copley JT, Glover AG et al. (2011) Community dynamics over 14 years at the Eiffel Tower hydrothermal edifice on the Mid-Atlantic Ridge. Limn Oceanogr: 56(5), 1624–1640.

[10] Tunnicliffe V, Garrett J, Johnson H (1990) Physical and biological factors affecting the behaviour and mortality of hydrothermal vent tubeworms (vestimentiferans). Deep Sea Res 37(1): 103–125.

[11] Copley JTP, Tyler PA, Van Dover CL, Schultz A, Dickson P, et al (1999) Subannual Temporal Variation in Faunal Distributions at the TAG Hydrothermal Mound (26° N, Mid-Atlantic Ridge). Mar Ecol 20(3–4): 291–306.

[12] Puillat I, Lanteri N, Drogou JF, Blandin J, Géli L et al. (2012) Open-Sea Observatories: A New Technology to Bring the Pulse of the Sea to Human Awareness. In: Marcelli M editor. Oceanography, ISBN: 978-953-51-0301-1, InTech 3–40.

[13] Devey CW, Fisher CR, Scott S (2007) Responisble science at hydrothermal vents. Oceanography 20(1): 162–171.

[14] Auffret Y, Sarrazin J, Coail JY, Delauney L, Legrand J et al. (2009) TEMPO-Mini: a custom-designed instrument for real-time monitoring of hydrothermal vent ecosystems. Martech 2009 conference proceedings.

[15] Sarrazin J, Blandin J, Delauney L, Dentrecolas S, Dorval P et al. (2007) TEMPO: A new ecological module for studying deep-sea community dynamics at hydrothermal vents. OCEANS ’07 IEEE, Aberdeen, June 2007. Proceedings no. 061215-042.

[16] Tivey MK, Delaney JR (1986) Growth of large sulfide structures on the Endeavour Segment of the Juan de Fuca Ridge. Earth Planet Sci Lett 77: 303–317.

[17] Sarrazin J, Juniper S (1999) Biological characteristics of a hydrothermal edifice mosaic community. Mar Ecol Progr Ser 185: 1–19.

[18] Urcuyo IA, Massoth GJ, Julian D, Fisher CR (2003) Habitat, growth and physiological ecology of a basaltic community of *Ridgeia piscesae* from the Juan de Fuca Ridge. Deep Sea Res Pt I 50(6): 763–780.

[19] Robert K, Onthank KL, Juniper SK, Lee RW (2012) Small-scale thermal responses of hydrothermal vent polynoid polychaetes: Preliminary in situ experiments and methodological development. J Exp Mar Biol Ecol 420–421: 69–76.

[20] Vuillemin R, Le Roux D, Dorval P, Bucas K, Sudreau JP, et al (2009) CHEMINI: A new in situ CHEmical MINIaturized analyzer. Deep Sea Res Pt I 56(8): 1391–1399.

[21] Rasband WS (2012) ImageJ, U.S. National Institutes of Health, Bethesda, Maryland, USA, http://imagej.nih.gov/ij/, 1997–2012.

[22] Warnes GR (2012) Includes R source code and/or documentation contributed by: B Bolker, L Bonebakker, R Gentleman, W Huber A Liaw, T Lumley, M Maechler, A Magnusson, S Moeller, M Schwartz and B Venables. gplots: Various R programming tools for plotting data. R package version 2.11.0. http://CRAN.R-project.org/package=gplotsRasband, W.S., 2012. ImageJ, U. S. National Institutes of Health, Bethesda, Maryland, USA, http://imagej.nih.gov/ij/, 1997–2012.

[23] Zambrano-Bigiarini M (2012) hydroTSM: Time series management, analysis and interpolation for hydrological modelling. R package version 0.3–6. Available: http://CRAN.R-project.org/package=hydroTSM.

[24] R Core Team (2012) R: A language and environment for statistical computing. R Foundation for Statistical Computing, Vienna, Austria. ISBN 3-900051-07-0, URL http://www.R-project.org/.

[25] De la Cruz M (2008) Metodos para analizar datos puntuales. In: Introduccion al Analisis Espacial de Datos en Ecologia y Ciencias Ambientales: Metodos y Aplicaciones (eds. Maestre FT, Escudero A, Bonet A 76–127. Asociacion Espanola de Ecologia Terrestre, Universidad Rey Juan Carlos y Caja de Ahorros del Mediterraneo, Madrid. ISBN: 978-84-9849-308-5.

[26] Legendre P (2012) Whittaker-Robinson periodogram. R program and documentation available: www.numericalecology.com.

[27] Legendre P, Legendre L (2012) Numerical ecology. Third English Edition. Elsevier Ed. p. 306.

[28] Borcard D, Legendre P, Drapeau P (1992) Partialling out the spatial component of ecological variation. Ecology 73: 1045–1055.

[29] Peres-neto PR, Legendre P, Dray S, Borcard D (2006) Variation partitioning of species data matrices: Estimation and comparison of fractions. Ecology 87(10): 2614–2625. 17089669 10.1890/0012-9658(2006)87[2614:vposdm]2.0.co;2

[30] Borcard D, Gillet F, Legendre P (2011) Numerical ecology with R. Use R! series, Springer Science, New York. p. 306.

[31] Dixon PM (2002) Nearest-neighbor contingency table analysis of spatial segregation for several species. Ecoscience 9 (2): 142–151.

[32] Bright M, Lallier FH (2010) The biology of Vestimentiferan tubeworms. Oceanogr Mar Biol, Annu Rev 48(2010): 213–266.

[33] Andersen AC, Flores JF, Hourdez S (2006) Comparative branchial plume biometry between two extreme ecotypes of the hydrothermal vent tubeworm *Ridgeia piscesae.* . Can J Zool 1822 1810–1822.

[34] Chevaldonné P, Desbruyères D, Haitre M (1991) Time-series of temperature from three deep-sea hydrothermal vent sites. Deep Sea Res 38(11): 1417–1430.

[35] Van Dover CL (1994) In situ spawning of hydrothermal vent tubeworms (*Riftia pachyptila*). Biol Bull 186: 134–135. 29283302 10.2307/1542043

[36] MacDonald IR, Tunniclife V, Southward EC (2002) Detection of sperm transfer and synchronous fertilization in Ridgeia piscesae at the Endeavour Segment, Juan de Fuca Ridge. Cah Biol Mar 43: 395–398.

[37] Desbruyeres D, Segonzac M, Bright M (Eds.) (2006) Handbook of Deep-sea Hydrothermal Vent Fauna. Second Completely Revised Edition. Denisia, 18. Biologiezentrum der Oberösterreichischen Landesmuseen, Linz, Austria. 544p.

[38] Levesque C, Juniper SK, Limen H (2006) Spatial organization of food webs along habitat gradients at deep-sea hydrothermal vents on Axial Volcano, Northeast Pacific. Deep Sea Res Pt I 53(4): 726–739.

[39] Britayev TA, Doignon G, Eeckhaut I (1999) Symbiotic polychaetes from Papua New Guinea associated with echinoderms, with descriptions of three new species. Cah Biol Mar 40: 359–374.

[40] Struck TH, Purschke G, Halanych KM (2005) A scaleless scale worm: Molecular evidence for the phylogenetic placement of Pisione remota (Pisionidae, Annelida) Mar Biol Res. 1(4): 243–253.

[41] Govenar BW, Bergquist DC, Urcuyo IA, Eckner JT, Fisher CR (2002) Three *Ridgeia piscesae* assemblages from a single Juan de Fuca Ridge sulphide edifice. Cah Biol Mar 43: 247–252.

[42] Tsurumi M, Tunnicliffe V (2003) Tubeworm-associated communities at hydrothermal vents on the Juan de Fuca Ridge, northeast Pacific. Deep Sea Res Pt I 50(5): 611–629.

[43] Bamber RN (2009) Two new species of *Sericosura* Fry & Hedgpeth, 1969 (Arthropoda: Pycnogonida: Ammotheidae), and a reassessment of the genus. Zootaxa 68: 56–68. 10.11646/zootaxa.3669.2.8 26312332

[44] Bain AB, Govedich FG (2004) Courtship and mating behaviour in the Pycnogonida (Chelicerata: Class Pycnogonida): a summary. Invert Reprod Dev 43: 63–79.

[45] Bergquist DC, Eckner JT, Urcuyo IA, Cordes EE, Hourdez S, et al (2007) Using stable isotopes and quantitative community characteristics to determine a local hydrothermal vent food web. Mar Ecol Prog Ser 330(1): 49–65.

[46] Martell KA, Tunnicliffe V, Macdonald IR (2002) Biological features of a buccinid whelk (Gastropoda, Neogastropoda) at the Endeavour vent fields of juan de Fuca Ridge, Northeast Pacific. J Molluscan Stud 68: 45–53.

[47] Aguzzi J, Costa C, Furushima Y, Chiesa J (2010) Company J et al (2010) Behavioral rhythms of hydrocarbon seep fauna in relation to internal tides. Mar Ecol Prog Ser 418: 47–56.

[48] Tunnicliffe V, Jensen RG (1987) Distribution and behaviour of the spider crab *Macroregonia macrochira* Sakai (Brachyura) around the hydrothermal vents of the northeast Pacific. Can J Zool 65: 2442–2449.

[49] Martin JW, Pettit G (1998) *Caprella bathytatos* New Species (Crustacea, Amphipoda, Capresslidae), from the Mouthparts of the Crab *Macroregonia macrochira* Sakai (Brachyura, Majidae) in the Vicinity of Deep-sea Hydrothermal vents off British Columbia. B Mar Sci 63: 189–198.

[50] Sarrazin J, Juniper SK, Massoth G, Legendre P (1999) Physical and chemical factors influencing species distributions on hydrothermal sulfide edifices of the Juan de Fuca Ridge, northeast Pacific. Mar Ecol Prog Ser 190: 89–112.

[51] Le Bris N, Govenar BW, Legall C, Fisher C (2006) Variability of physico-chemical conditions in 9°50′N EPR diffuse flow vent habitats. Mar Chem 98(2–4): 167–182.

[52] Hautala S, Johnson HP, Pruis M, García-Berdeal I, Bjorklund T (2012) Low-temperature hydrothermal plumes in the near-bottom boundary layer at Endeavour Segment, Juan de Fuca Ridge. Oceanography 25: 192–195.

[53] Thomsen L, Barnes C, Best M, Chapman R, Pirenne B, et al (2012) Ocean circulation promotes methane release from gas hydrate outcrops at the NEPTUNE Canada Barkley Canyon node. Geophys Ress Lett 39: L16605.

[54] Juniper SK, Matabos M, Mihály S, Ajayamohan RS, Gervais F, et al (2013) A year in Barkley Canyon: A time-series observatory study of mid-slope benthos and habitat dynamics using the NEPTUNE Canada network. Deep Sea Res Pt II 92: 114–123.

[55] Tivey MK, Bradley AM, Joyce TM, Kadko D (2002) Insights into tide-related variability at seafloor hydrothermal vents from time-series temperature measurements. Earth Planet Sci Lett 202: 693–707.

[56] Aguzzi J (2003) Company JB, Sardà F, Abelló P (2003) Circadian oxygen consumption patterns in continental slope *Nephrops norvegicus* (Decapoda: Nephropidae) in the western Mediterranean. J. Crustacean Biol. 23: 749–757.

[57] Delaney JR, Kelley DS, Lilley MD, Butterfield DA, McDuff RE et al. (1997) The Endeavour Hydrothermal System I: Cellular circulation above an active cracking front yields large sulfide structures, “fresh” vent water, and hyperthermophilic archae, RIDGE Events: 11–19.

