# Supplementary figures and images for: Rhythms and Community Dynamics of a Hydrothermal Tubeworm Assemblage at Main Endeavour Field – A Multidisciplinary Deep-Sea Observatory Approach
Source: PLoS One. 2014 May 8;9(5):e96924. doi: 10.1371/journal.pone.0096924 (PMC4014580; doi:10.1371/journal.pone.0096924)

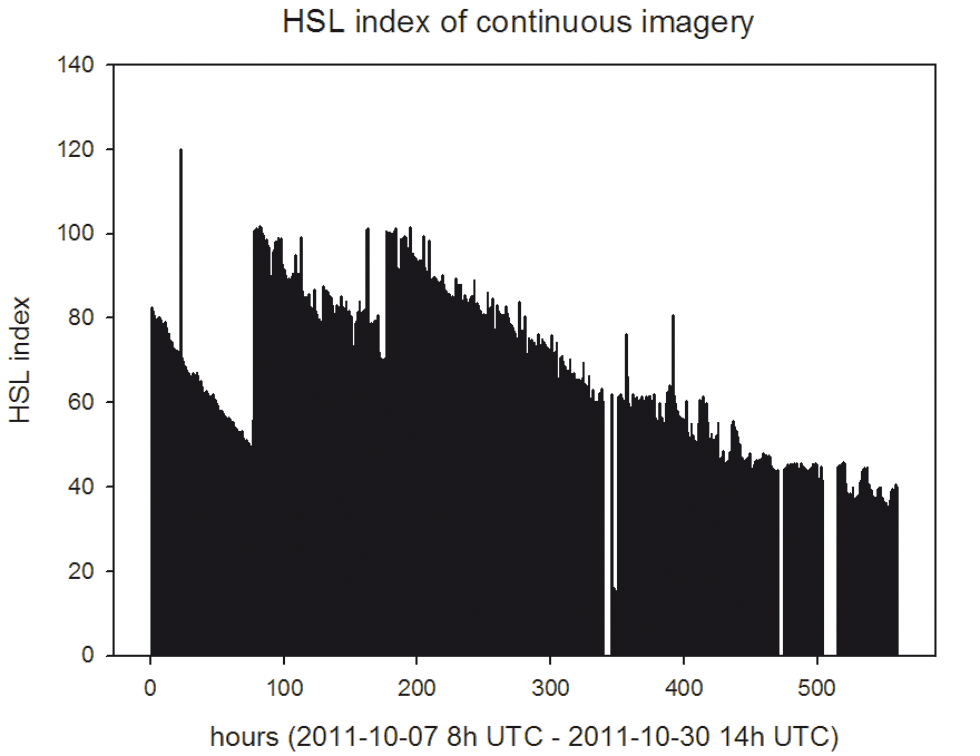

Supplement: Figure S1 — HSL (hue, saturation and light) index of imagery recorded. HSL index, combining hue, saturation and light, was calculated to assess the quality of the hourly imagery recorded during the continuous recording period (2011-10-07 to 2011-10-30). The HSL index, whose maximum value is 255 (white) and minimum is 0 (black), shows the general darkening of the recorded imagery with time. (TIF) [file pone.0096924.s001.tif]

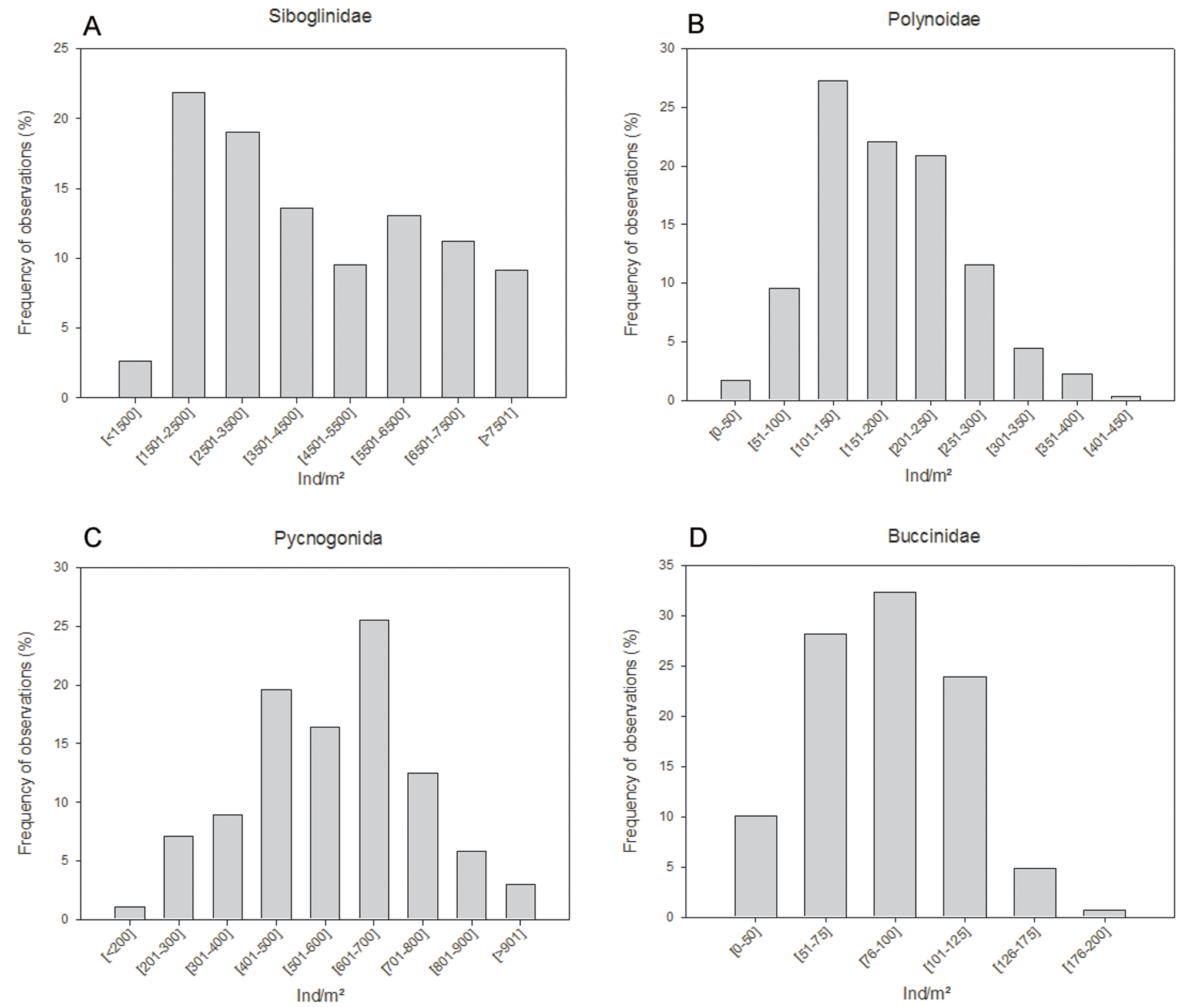

Supplement: Figure S2 — Frequency of faunal density observations per taxon. Percentage of the images observed with faunal densities in defined categories. N = 559-23 gaps, i.e. 536 images analysed. (TIF) [file pone.0096924.s002.tif]

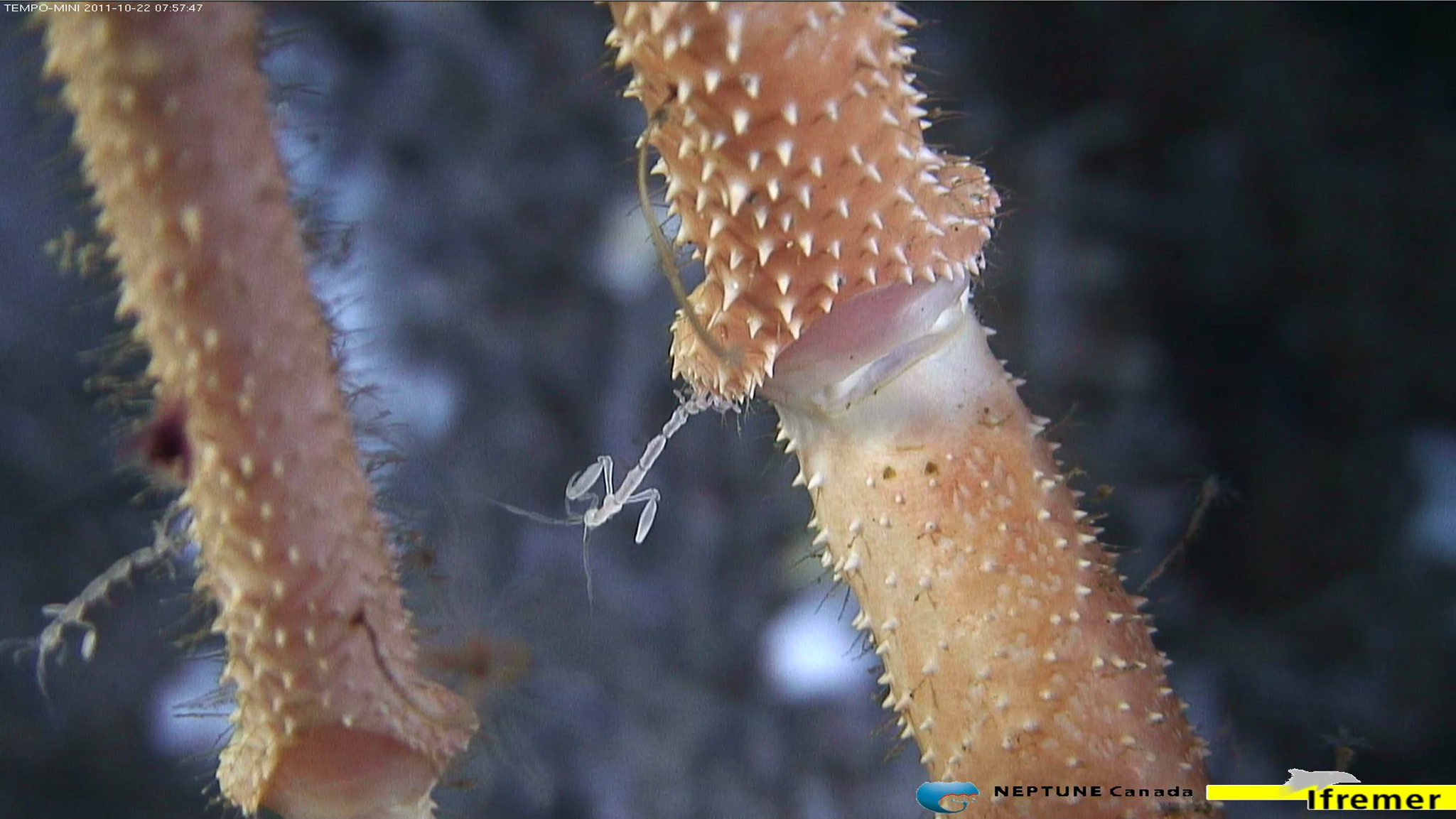

Supplement: Figure S3 — Spider crab on top of TEMPO-mini module. A majid spider crab, probably Macroregonia macrochira, sitting on top of the TEMPO-mini module. Two caprellid individuals attached to its legs can be recognised (possibly Caprella bathytatos, Caprellidae, Amphipoda). (TIF) [file pone.0096924.s003.tif]
